# Large uncertainties in trends of energy demand for heating and cooling under climate change

**DOI:** 10.1038/s41467-021-25504-8

**Published:** 2021-08-31

**Authors:** Adrien Deroubaix, Inga Labuhn, Marie Camredon, Benjamin Gaubert, Paul-Arthur Monerie, Max Popp, Johanna Ramarohetra, Yohan Ruprich-Robert, Levi G. Silvers, Guillaume Siour

**Affiliations:** 1 LMD - IPSL, École Polytechnique, Institut Polytechnique de Paris, ENS, IPSL Research University, Sorbonne Université, CNRS, Palaiseau, France; 2 Institute of Geography, University of Bremen, Bremen, Germany; 3 LISA, Université Paris-Est Créteil, CNRS, Université de Paris, IPSL, Créteil, France; 4 Atmospheric Chemistry Observations & Modeling Laboratory (ACOM), National Center for Atmospheric Research, Boulder, CO USA; 5 Department of Meteorology, National Centre for Atmospheric Science, University of Reading, Reading, UK; 6 Independent researcher, Barcelona, Spain; 7 Barcelona Supercomputing Center, BSC, Barcelona, Spain; 8 School of Marine and Atmospheric Sciences, State University of New York at Stony Brook, Stony Brook, NY USA

**Keywords:** Climate-change impacts, Energy supply and demand

## Abstract

The energy demand for heating and cooling buildings is changing with global warming. Using proxies of climate-driven energy demand based on the heating and cooling Degree-Days methodology applied to thirty global climate model simulations, we show that, over all continental areas, the climate-driven energy demand trends for heating and cooling were weak, changing by less than 10% from 1950 to 1990, but become stronger from 1990 to 2030, changing by more than 10%. With the multi-model mean, the increasing trends in cooling energy demand are more pronounced than the decreasing trends in heating. The changes in cooling, however, are highly variable depending on individual simulations, ranging from a few to several hundred percent in most of the densely populated mid-latitude areas. This work presents an example of the challenges that accompany future energy demand quantification as a result of the uncertainty in the projected climate.

## Abstract (web summary)

The energy demand for heating and cooling buildings is changing with global warming. Here the authors show that trends in cooling energy demands are increasing, although the magnitude is extremely uncertain, which highlights challenges for future energy demand quantification.

## Introduction

In a warming world, most regions are expected to experience a reduction in the energy needed for heating, and an increase in the energy needed for cooling buildings^1^. Anticipating those changes will help communities to adapt their buildings and energy systems to future climate. The energy demand for heating and cooling buildings is driven by a climatic component, a socio-economic component (population density and behavior of people, gross domestic product, price of energy) and by a technological component (design and material determining the thermal properties of the building, efficiency of heating and cooling systems)^2–4^. In addition to long-term trends in these three components, there is a short-term variability in energy demand, and in related CO_2_ emissions, which is mostly linked to climate variability^4,5^.

Among the climate variables that influence the energy demand, ambient temperature is prominent^6^, or more precisely its combination with humidity^7^. The minimum and maximum daily temperatures are good predictors of the energy demand^8^ as they represent the diurnal cycle of ambient temperature. The amplitude of this diurnal cycle is large in dry areas and small in wet areas. The day-to-day variability in energy demand depends on temperature following a V-shape curve with a minimum related to human thermal comfort as well as other socio-economic and technological factors^9,10^. This minimum is found for a similar daily mean temperature around 16 °C for 35 countries in Europe^11^. Therefore, a comprehensive analysis relating the trends in the projected temperature and its consequences on energy demand is possible.

The Degree-Days methodology is the historical method for estimating the heating and cooling energy demand of buildings^12–14^ (cf. Methods section). A key assumption of this method is that the average temperature of a day provides a good proxy for the human thermal discomfort^13,14^, and thus of the daily energy demand^9,12,15^. Degree-Days represent the difference between the outside daily temperature and the range of comfortable indoor temperatures. In other words, Degree-Days are the cumulated temperature during one day below a base temperature, the so-called Heating Degree-Days (HDD); and above a base temperature, the so-called Cooling Degree-Days (CDD). In the context of climate change^16^, the estimation of energy demand of buildings in the coming decades should include changes in climate-driven energy demand, which are expected to become increasingly important in the future. The use of climate projections for this purpose is thus pivotal. Future changes in the energy demand for heating and cooling buildings through the twenty-first century have been estimated using Degree-Days calculated with the temperature output from climate model simulations for the US^15,17^ and Europe^18^ individually.

A vast amount of literature investigates climate change impacts on future energy demand together with implications for the society in terms of energy system capacity^19^, regulations and mitigation^20^, adaptation^21^ or socio-economic developments^22^. These studies take into account the complexity of the socio-economic and technological components but the climatic component is overly simplified. For instance, the use of multi-model mean (MMM) climate projections or a single scenario of greenhouse-gas emissions neglects the full range of possible future temperatures. A recent global analysis of numerous factors involved in the energy demand predictions showed a weak agreement in these projections for hot and cold days^23^. To go further, the future temperature being highly variable among climate projections^24,25^, the uncertainties related to climate need to be quantified and included in estimates of future energy demand. A consistent global analysis of these uncertainties is still missing.

This study focuses on proxies of the climate-driven energy demand for heating and cooling buildings, and presents a global analysis of future trends together with a comprehensive analysis of uncertainties linked to temperature projections. To this end, the proxies of the climate-driven energy demand derived from the Degree-Days methodology are calculated using the simulated surface air temperatures of 30 CMIP5 (Coupled Model Intercomparison Project phase 5) general circulation models (GCMs)^26^ and two pathways of future greenhouse-gas concentrations^27^. We show that the increasing trends in cooling energy demand are stronger than the decreasing trends in heating with the MMM of all 30 models. However, where the trends in cooling are the strongest, the variability of the trends between individual models is high, making estimates of future energy demand uncertain in these regions.

## Results

### Proxies of climate-driven energy demand

HDD and CDD calculated with the temperature of historical climate simulations have been validated against observations^17,18^. We define our heating and cooling—climate-driven energy demand—proxies as the annual HDD and CDD sums calculated from daily mean, minimum and maximum temperatures following the UK Met Office methodology (Table 1, Methods section) for each of the 30 CMIP5 climate simulations (Supplementary Table 2). The advantage of HDD or CDD annual sums is that they can be compared on a global scale, regardless of the timing and length of local heating and cooling seasons. The heating and cooling proxies are presented for the MMM as averages over three 20-year periods 1941–1960, 1981–2000 and 2021–2040 (Supplementary Fig. 1).

**Table 1:** Calculation of Heating Degree-Days (HDD) and Cooling Degree-Days (CDD) according to the UK Met Office for a day defined with a daily mean (Tmean), minimum (Tmin) and maximum (Tmax) surface air temperature.

Condition on temperature	HDD UK calculation (Tbase = 15.5 °C)	Interpretation
	CDD UK calculation (Tbase = 22 °C)	
Tmax ≤ **Tbase**	HDD = **Tbase** − Tmean	Cold day
	CDD = 0	Cold day
Tmean ≤ **Tbase** < Tmax	HDD = (**Tbase** − Tmin)/2 − (Tmax − **Tbase**)/4	Mostly cold day
	CDD = (Tmax − **Tbase**)/4	Mostly cold day
Tmin < **Tbase** < Tmean	HDD = (**Tbase** − Tmin)/4	Mostly warm day
	CDD = (Tmax − **Tbase**)/2 − (**Tbase** − Tmin)/4	Mostly warm day
Tmin ≥ **Tbase**	HDD = 0	Warm day
	CDD = Tmean − **Tbase**	Warm day

HDD is calculated with a base temperature (Tbase) of 15.5 °C, and CDD with a base temperature of 22 °C.

The spatial patterns of the MMM of the heating and cooling proxies are closely linked to the MMM of temperature on a global scale (comparing Supplementary Fig. 1 and Supplementary Fig. 2). The decrease in HDD and the increase in CDD between the three studied time periods are also consistent with the underlying temperature increase. Our results show typical values of the heating proxy over land areas between 0 and 1500 HDD in inter-tropical regions (from 30°N to 30°S), between 1500 and 5000 HDD in mid-latitude regions (from 60°N to 30°N; or from 60°S to 30°S) and above 5000 HDD in polar regions (above 60°N or 60°S). Values of the cooling proxy are between 400 and 2000 CDD in inter-tropical regions, and between 0 and 400 CDD in mid-latitudes. These values change in a warming world. However the magnitude of the changes is not globally uniform (Supplementary Fig. 1).

### Heating and cooling changes in the past and in the future

To quantify the magnitude of past and future changes, we use the absolute differences in the heating and cooling proxies between 1981–2000 and 1941–1960, henceforth referred to as *past* changes, and between 2021–2040 and 1981–2000, henceforth referred to as *future* changes. We estimate the heating and cooling proxies from the CMIP5 historical simulations for the *past* and from the projections using the Representative Concentration Pathway 8.5^27^ (RCP8.5; unless otherwise stated) for the *future*. The MMM heating proxy decreased and the MMM cooling proxy increased over the course of both the *past* and the *future* time periods.

**Fig. 1:**
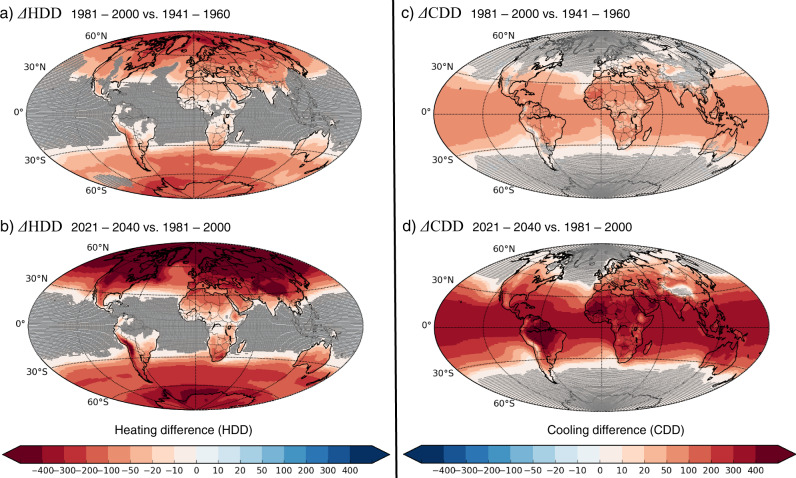
Global climate-driven changes in energy demand for heating and cooling buildings. The quantification of the change is expressed as absolute differences in annual (left) Heating Degree-Days (ΔHDD) and (right) Cooling Degree-Days (ΔCDD) between the periods (**a, c**) 1981–2000 and 1941–1960, and (**b, d**) 2021–2040 and 1981–2000. Multi-model means of annual HDD and CDD sums were calculated using the daily mean, minimum and maximum temperatures from 30 CMIP5 models, and averaged over the 20-year periods. The areas shaded in gray indicate locations where the difference is not significantly different from zero according to Student *t* test at a 95% confidence interval. Projections are based on the RCP8.5 scenario. Note that the color scale in (**a** and **b**) is inverted compared to (**c** and **d**), so that in all panels red colors correspond to changes caused by increasing temperatures.

In the *past*, the most important changes in the heating proxy (below −200 HDD) occurred over polar regions (Fig. 1a), while, in the *future*, a decrease in the heating proxy of at least this magnitude occurs over the entire Northern Hemisphere (Fig. 1b). The increase in the cooling proxy was small in the *past*, below +100 CDD, everywhere except in some (semi-)arid parts of West Africa (Fig. 1c). The projected *future* increase in the cooling proxy, on the other hand, exceeds +100 CDD in most of the mid-latitude regions, exceeds +300 CDD in large parts of the tropics, and exceeds +400 CDD in Amazonia, in parts of the Sahel and in the Arabian Peninsula (Fig. 1d).

Changes in the heating and cooling proxies have similar spatial patterns in the *past* and the *future*, with an overall extension of the areas with significant changes projected for the *future* (Fig. 1a, c compared to Fig. 1b, d). Mid-latitude regions present significant changes in both the heating and the cooling proxies. Areas with non-significant changes in the heating proxy are projected to reduce to tropical ocean regions, including tropical islands, as well as Amazonia in the *future* (Fig. 1b). Conversely, areas with non-significant changes in the cooling proxy are projected to reduce to the northern (above 40°N) and southern (below 40°S) oceans, whereas there is a significant change over all continental areas (except Greenland and Antarctica) in the *future* (Fig. 1d).

### Comparing trends in heating and cooling

Even when the absolute differences in some regions are small from one period to another, they could lead to significant changes in societal behavior, such as widespread acquisition of cooling systems^21^, as people feel a difference in thermal comfort relative to the past. We quantify trends in climate-driven energy demand for heating and cooling buildings by computing the relative differences in our proxies for the *past* and the *future* (cf. Methods section), which leads to important trends in the surroundings of the areas with non-significant changes (i.e., gray shaded areas in Fig. 1).

**Fig. 2:**
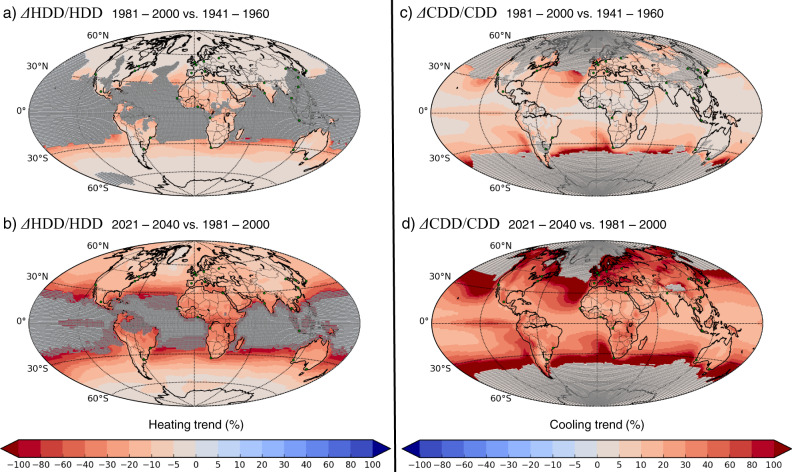
Global climate-driven trends in energy demand for heating and cooling buildings. Trends are expressed in Heating Degree-Days (ΔHDD/HDD) and Cooling Degree-Days (ΔCDD/CDD) between the periods (**a, c**) 1981–2000 and 1941–1960, and (**b, d**) 2021–2040 and 1981–2000. Multi-model means of annual HDD and CDD sums were calculated using the daily mean, minimum and maximum temperatures from 30 CMIP5 simulations, and averaged over the 20-year periods. Differences are given in % compared to the earlier period. The areas shaded in gray indicate locations where the difference is not significantly different from zero according to Student *t* test at a 95% confidence interval. Projections are based on the RCP8.5 scenario. Note that the color scale in (**a** and **b**) is inverted compared (**c** and **d**), so that in all panels red colors correspond to changes caused by increasing temperatures. Green dots denote the location of the cities in Fig. 3 of the main text and Supplementary Table 1.

Over continental areas, the decreasing trend in the MMM heating proxy was weak, ranging from −20 to 0% in the *past* (Fig. 2a). This trend is projected to become clearly negative everywhere in the *future*, reaching at least −5% (Fig. 2b).

The increasing trend in the MMM cooling proxy was weak in the *past*, ranging between 0 and +20% over continental areas (Fig. 2c). This trend is also projected to be more pronounced in the *future*, exceeding +10% everywhere, reaching at least +20% over mid-latitude regions, and more than +60% in many northern hemisphere regions (Fig. 2d). Over mid-latitude oceans, the projected trend in the cooling proxy is to exceed +100%, which leads to strong gradients close to the coastlines, where an important part of the population lives.

### Uncertainty from inter-model variability

The MMM must be interpreted with caution^28^, as the variability in simulated surface air temperature between individual models can be large^24^. We select major densely populated areas worldwide to analyze the robustness of the aforementioned MMM results across the thirty simulations. We focus on grid cells which contain (mega)-city locations to analyze the inter-model variability in the heating and cooling proxy trends in the *past* and in the *future*.

**Fig. 3:**
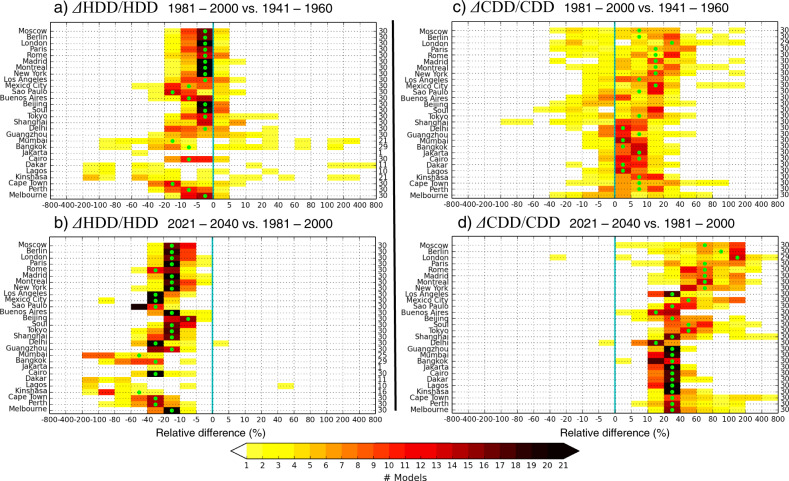
Inter-model variability of climate-driven trends in energy demand for heating and cooling buildings at selected cities. Trends are expressed as relative differences in annual Heating Degree-Days (HDD) and Cooling Degree-Days (CDD), calculated for each of the 30 CMIP5 simulations. Relative difference in HDD (ΔHDD/HDD) between the (**a**) 1981–2000 and 1941–1960 averages, and (**b**) the 2021–2040 and 1981–2000 averages; relative difference in CDD (ΔCDD/CDD) between the (**c**) 1981–2000 and 1941–1960 averages, and the (**d**) 2021–2040 and 1981–2000 averages. Differences are given in % relative to the earlier period. Projections are based on the RCP8.5 scenario. The color bar denotes the number (#) of models in each range of relative change. The multi-model mean falls in the range marked by the green dots. Numbers on the right indicate the number of models for which the difference between the two periods is significant. The vertical blue line marks zero.

In mid-latitude cities, there is solid consensus among the model simulations (more than 20 simulations of the 30 agree on the sign of change) in the estimation of the decreasing trend in the heating proxy, as evidenced by the small inter-model variability ranging from −20 to +10% in the *past* (Fig. 3a). This trend becomes negative in the *future* for all simulations, ranging from −60 to 0% (Fig. 3b). In tropical cities, even if the negative trend is weak, the inter-model variability was large in the *past* (with no consensus among the simulations) and becomes smaller (overall consensus) in the *future*.

The inter-model variability in the increasing trend of the cooling proxy is smaller in tropical cities than in mid-latitude cities in the *past* and in the *future*. In tropical cities, the increasing trend in the cooling proxy was weak in the *past* (about + 5% for the MMM) and associated with a small inter-model variability ranging from about −5% to +40% (Fig. 3c). For most of the tropical cities studied, there is a consensus not to simulate any trend (close to 0%) in the *past*. In the *future*, the projected trend in the cooling proxy is stronger (about + 30% for the MMM) and also associated with a small variability between simulations of about +10% to +60% (Fig. 3d). Consequently, the projected increasing trend in cooling over tropical regions is robust.

In mid-latitude cities, where the cooling is generally low, the increasing trend in the cooling proxy was weak in the *past* (about + 15% in the MMM) and associated with a large inter-model variability (Fig. 3c), ranging from moderate negative trends (about −20%) to strong positive trends (about + 60%). In the *future*, the increasing trend in the cooling proxy becomes stronger, with the MMM near +70% for most cities (Fig. 3d). There is a consensus among the simulations to predict an increase exceeding +10%. Nevertheless, the inter-model variability is large, with a trend in the cooling proxy reaching up to +400% in some cities. Consequently, a robust increase in the need for cooling over mid-latitude cities is predicted, but the quantification is highly uncertain.

### Uncertainty from future emission pathways

The uncertainty of anthropogenic emission pathways and of climate projections both contribute to the wide range of projections of future climate-driven energy demand for heating and cooling buildings. We study two pathway scenarios^16^, (i) business-as-usual and (ii) moderately mitigated, which are referred to as (i) RCP8.5 and (ii) RCP4.5. To investigate the impact of future greenhouse-gas emissions, we compare the trends in the heating and cooling proxies for the two scenarios in the *near-future* (as in the previous sections), and by the *end of the century*, the centennial trend (using the period 2081–2100 instead of 2021–2040 to compare with 1981–2000, cf. Supplementary Material Section II).

The difference between temperature projections based on RCP8.5 and RCP4.5 is small in the *near-future*. As a result, the magnitude of the trends in the MMM heating and cooling proxies, as well as the inter-model variability, are similar between the two scenarios (Supplementary Fig. 1 and comparing Supplementary Fig. 3 against Supplementary Fig. 4). As temperature projections based on RCP8.5 and RCP4.5 diverge during the twenty-first century, the question arises how scenario-dependent trends in heating and cooling relate to the uncertainty coming from the inter-model variability.

The projected centennial trends in the heating proxy for mid-latitude cities are similar for both RCP scenarios by the *end of the century*, ranging from (i) −20 to −80% for RCP8.5 compared to (ii) −10 to −60% for RCP4.5 (comparing Supplementary Fig. 5 against Supplementary Fig. 6). The decreasing trend in the heating proxy is therefore robust and comparable for both scenarios.

The projected trends in the cooling proxy for tropical cities are also robust and comparable between the scenarios when considering the *near-future*, but there is an important increase in the model variability by the *end of the century*, ranging from (i) + 40% to +200% for RCP8.5 compared to (ii) + 20% to +100% for RCP4.5. In mid-latitude cities, the quantification of the centennial trends in the cooling proxy becomes highly uncertain, but there is a consensus among the simulations to project a centennial trend greater than (i) + 200% for RCP8.5 and (ii) + 100% for RCP4.5. However, by the *end of the century*, the trends projected by individual simulations between RCP8.5 and RCP4.5 overlap although the MMM trends are different.

### Uncertainty from the methodology

Several tests were performed to study the influence of alternatives in the methodology on the simulated trends in the heating and cooling proxies: (a) Changing the calculation method of Degree-Days (UK vs. US) (cf. Supplementary Information Section IIIa, Supplementary Figs 7 and 8); (b) Changing the spatial resolution of the multi-model grid used to calculate HDD and CDD from 1° × 1° to 2° × 2° (cf. Supplementary Information Section IIIb, Supplementary Fig. 9); (c) Changing the temporal averaging periods from 20-year to 30-year averages (cf. Supplementary Information Section IIIc, Supplementary Fig. 10); (d) Correcting model biases based on the difference in monthly mean temperature of historic simulations and observations^29^ for the reference period 1981–2000 before calculating HDD and CDD (cf. Supplementary Information Section IIId, Supplementary Figs. 11, 12 and 13); (e) Calculating HDD and CDD from MMM daily temperatures (cf. Supplementary Information Section IIIe, Supplementary Fig. 14).

For the five tests, similar results are obtained in terms of the spatial patterns and of the magnitudes of the trends for the MMM. The inter-model variability is also comparable, even when the biases in temperature simulation are corrected. Furthermore, we demonstrate that our results are not sensitive to the choice of base temperature in the Degree-Days calculation (cf. Supplementary Information Section IV). The difference of HDD (resp. CDD) calculated with different base temperatures are constant in time, which means that the trends are the same when comparing the three time periods (Supplementary Fig. 15). We conclude that the quantification of future trends in the heating and cooling proxies is uncertain due primarily to the large inter-model variability and not due to details of the methodology.

## Discussion

Our study represents a step toward a more accurate quantification of the future climate-driven energy demand for the heating and cooling of buildings by taking into account the uncertainties related to temperature projections. We show, at the global scale, that the energy demand trends for the next two decades are robust because all thirty CMIP5 simulations project a decreasing trend in heating and an increasing trend in cooling. However, the uncertainties of these trends differ between heating and cooling because the inter-model variability is small for heating but large for cooling.

We made use of a metric derived from cumulated surface temperature to estimate the trends of climate-driven energy demand. The surface temperature is the most important determining factor for this demand^6^. Atmospheric humidity is also a key factor^30^. Our methodology includes some dependence on humidity, through the use of the daily minimum and maximum temperatures. Nevertheless, further research is needed for an explicit inclusion of humidity in the estimation of the future energy demand. Other climatological factors, such as precipitation or wind, are important as well to estimate the future end-use energy demand^8^. However these factors are physically linked to the temperature change, therefore focusing on the projected temperature, our study constitutes a necessary first step.

Reliable information is needed by individuals, city planners, policy makers and companies to manage or mitigate future changes in energy demand. The trends in the heating and cooling needs of buildings are useful to indicate which regions will likely experience large changes, and thus benefit most from improvements in thermal insulation and the efficiency of heating/cooling systems. For example, we show that the increasing trend in cooling for Paris (most analyzed mid-latitude cities present comparable results) is projected to reach +80% in the MMM, leading to a potential massive adoption of cooling systems. However, this number alone is not sufficient to assess whether such measures will indeed be needed, because the increase may be as small as +2% or as large as +348% according to individual model estimates. The quantification of the increasing trend in cooling is thus highly uncertain and highlights the need to take the inter-model variability into account when designing adaptation plans, whether they concern architecture, the efficiency of climatization systems, or power generation and networks.

Our analysis demonstrates that the increasing trends in cooling are particularly uncertain close to the coast of the northern Atlantic Ocean. This is because the temperature increase in the Arctic region is amplified due to Arctic sea ice reduction^31^. Consequences are expected for northern mid-latitude weather^32,33^ but there is no consensus across CMIP5 simulations^34^. Narrowing uncertainties in the temperature projections is highly desirable for reliable estimates of future energy demand for the heating and cooling of buildings.

## Methods

### Proxies of climate-driven energy demand for heating and cooling buildings

Surface temperatures used to be monitored at a daily time step before the digitization of meteorological data. Consequently, methods to estimate energy demand have also been based on temperature data with a daily time step. The Degree-Days methodology has long been used to estimate heating and cooling energy demand^12^, and relies on the link between human discomfort sensation and temperature variability^13,14^. The main assumption of the Degree-Days methodology is that the annual cumulative temperature above a temperature threshold (called base temperature, Tbase) of the daily mean temperature only (that we refer to as US calculation, Table 2), or in combination with daily minimum and maximum temperatures (that we refer to as UK calculation, Table 1), is a good proxy of the climate-driven energy demand for heating and cooling buildings^12,15^. The UK calculation based on four day types (cold day, mostly cold day, mostly warm day or warm day*)* is more adequate to analyze energy demand in different climate regions^12,14^. This is because it takes into account differences in the amplitude of the diurnal temperature cycle. Although the integration of other climatic (cloud, wind, precipitation, snow) and non-climatic (socio-economical or technological) variables are needed to accurately estimate the end-use energy demand of buildings^7,8^, the temperature is the main climate driver of energy demand for building heating and cooling^6^. The analysis of HDD and CDD, includes the dimension of human perception of climate change.

**Table 2:** Calculation of Heating Degree-Days (HDD) and Cooling Degree-Days (CDD) according to the US ASHREA for a day defined with a daily mean (Tmean) surface air temperature.

Condition on temperature	HDD US calculation (Tbase = 18.3 °C)	Interpretation
	CDD US calculation (Tbase = 18.3 °C)	
Tmean ≤ **Tbase**	HDD = **Tbase** − Tmean	Cold day
	CDD = 0	Cold day
Tmean ≥ **Tbase**	HDD = 0	Warm day
	CDD = Tmean − **Tbase**	Warm day

HDD and CDD are calculated with a base temperature (Tbase) of 18.3 °C.

For the US calculation, we use a single Tbase of 18.3 °C for both heating and cooling, i.e., there is one threshold, and two conditions on temperature, above or below the threshold (Table 2). For the UK calculation, which includes the daily mean, minimum and maximum (instead of the mean only), we use 15.5 °C as Tbase for heating and 22 °C as Tbase for cooling^18^, i.e., there are two thresholds and four conditions on temperature (Table 1).

In the main article, we present our results with the UK calculation. In the supplement, we present our results again but with the US calculation, and we include a comparison of the trends estimated using both calculation methods (cf. Supplementary Information Section IIIa). In addition, as there are several alternative Tbase values applied for heating and cooling in the literature^18^, we perform a sensitivity test of the heating and cooling trends using 15.5 °C and 22 °C, as well as using only 18.3 °C with the UK calculation (cf. Supplementary Information Section IV).

### Application of the methodology to CMIP5 simulations

Changes in future energy demand related to temperature change can be estimated with the Degree-Days method using climate projections by GCMs. The available output from the 30 CMIP5 simulations (Supplementary Table 1) includes daily mean, minimum and maximum temperature necessary for Degree-Days calculations.

The historical simulations are used for the *past*, and two Representative Concentration Pathways (RCP4.5 and RCP8.5)^27^ are used to cover a wide range of possible greenhouse gas concentrations and the resultant temperature change in the *future*. We focus on 20-year time periods (defined as in IPCC 2013, AR5 WGI, Annex I): (i) 1941–1960, (ii) 1981–2000, and (iii) 2021–2040 (and (iv) 2081–2100 in Supplementary Information Section II).

Although only inhabited land areas are relevant for heating and cooling, we include all land and ocean areas in the analysis to better understand the underlying global climate patterns, and because they are relevant for islands.

Our general methodology is described in the following five steps. Each step is applied to all grid cells:

1. Daily HDD and CDD are calculated with the UK Met Office equations (Table 1).
2. Annual sums of HDD and of CDD are calculated.
3. For the three 20-year time periods, the annual sums of HDD and CDD are averaged for each simulation at the native spatial resolution of the model.
4. All HDD and CDD 20-year means are interpolated spatially to a 1° by 1° grid. Python interpolation tools with a cubic method (using the function *scipy.interpolate.interp2d*) are used to avoid abrupt changes from one grid cell to another.
5. The means for the 20-year time period from each model are averaged in each grid cell to compute the MMM of HDD and CDD. These means are shown in Supplementary Fig. 1.

In order to compare the spatial patterns of HDD and CDD with the one of temperature, we use the same methodology with annual averages of surface air temperature for the three time periods (Supplementary Fig. 1g-i) in order to calculate the MMM of surface air temperature in Supplementary Information Section I.

We investigate two RCPs (4.5 and 8.5)^27^ proposed by the CMIP5 simulations^26^ and two future periods (i) 2021–2040 and (ii) 2081–2100 (modifying Step 1) in Supplementary Information Section II.

In addition to the general methodology described by the five steps above, we list particular alternative methods below that have been used to test the robustness of the proxies of climate-driven trends in the energy demand:

- To test if the results are influenced by the Degree-Days calculation method, we compare the two most widely used approaches, the UK Met Office calculation (applied to obtain our results presented in the main article) and a simpler calculation used in the USA. We use HDD and CDD with US ASHREA (American Society of Heating, Refrigerating and Air-Conditioning Engineers) equations (Table 2) instead of the UK Met Office (modifying Step 1) in Supplementary Information Section IIIa.
- To test the influence of the spatial interpolation, we reduce the spatial resolution of the grid from 1° by 1° (applied to obtain our results presented in the main article) to 2° by 2°. We interpolate on a 2° grid (modifying Step 4) in Supplementary Information Section IIIb.
- To test the influence of the length of the time span over which HDD and CDD are averaged, we increase the length of the time spans over which annual HDD and CDD are averaged from 20 years (applied to obtain our results presented in the main article) to 30 years in Supplementary Information Section IIIc.
- To test the influence of the systematic biases that appears in some regions for some models compared to observations, we correct the model biases based observations of the CRU TS 4.0 data set at 0.5° resolution^29^ in Supplementary Information Section IIId.
- To test the influence of applying the MMM on daily temperature instead of on HDD and CDD, we calculate HDD and CDD from a MMM of daily temperature (modifying Step 1) in Supplementary Information Section IIIe. Note that this method leads to a single value of HDD and CDD (instead of the 30 for the other tests).

### Calculation of trends

The absolute differences in HDD and CDD values (i.e., the annual HDD and CDD sums averaged over 20 years) are calculated between the 1981–2000 and 1941–1960 averages, referred to as *past* changes, and between the 2021–2040 and 1981–2000 averages, referred to as *future* changes, for each model and for the MMM (Fig. 1). Heating and cooling trends are expressed in terms of relative HDD and CDD differences, in percentage compared to the earlier period, shown for the MMM (Fig. 2). A Student *t* test (using two dependent data samples) is applied to test whether the averages of the thirty simulations are significantly different over two compared time periods at a 95% confidence interval, assuming that the values of the thirty simulations are normally distributed in each grid cell.

In order to compute the relative differences in HDD and CDD values, i.e., our proxy of the climate-driven energy demand trends, two conditions are used:

- The absolute differences in HDD and CDD values must be significant according the statistical test;
- The annual average in HDD and CDD values for the earlier period which is the denominator of the relative differences, must be >1.

Conceptually, our methodology allows comparing the climate-driven energy demand for heating or for cooling of an exact same building (structure, materials, heating, and cooling system) in different locations of the world. For a specific location, the trends in HDD and in CDD represent how the energy demand of this hypothetical building is projected to change in time due to the climate.

### Reporting summary

Further information on research design is available in the Nature Research Reporting Summary linked to this article.

## Supplementary information

Supplementary Information File

Peer Review File

Reporting Summary

## Data Availability

The temperature simulation data used in this study are available under the accession code https://esgf-node.ipsl.upmc.fr/search/cmip5-ipsl/.

## Code availability

The custom code, written in Python, to reproduce the analyses and figures of this study are available from the authors upon reasonable request.
