# Supplementary material for: Large uncertainties in trends of energy demand for heating and cooling under climate change
Source: Nat Commun. 2021 Aug 31;12:5197. doi: 10.1038/s41467-021-25504-8 (PMC8408235; doi:10.1038/s41467-021-25504-8)
Supplement: Supplementary file 1 — Supplementary Information File [file 41467_2021_25504_MOESM1_ESM.pdf]

Supplementary Information to

Deroubaix et al.,

Large uncertainties in trends of energy demand for heating and cooling under climate change

I. Global patterns of HDD and CDD in comparison with temperature

The global HDD patterns (Supplementary Figure 1 a, b, c) correspond to three main climatic zones, the polar regions above 60°N and 60°S (HDD > 5000), the mid-latitude regions from 60°N to 30°N and from 60°S to 30°S (1000 < HDD < 5000) and the tropical regions between 30°N and 30°S (HDD < 1000). Regarding the global CDD patterns (Supplementary Figure 1 d, e, f), the highest values are observed in subtropical deserts (CDD > 2000), over tropical oceans (1500 < CDD < 2000), and in tropical regions such as India and Amazonia (CDD > 2000). Over mid-latitude regions, there is an important variability along the same latitude, *e.g.* CDD in the eastern USA (ranging from 400 to 1100) is much higher than in Europe (ranging from 0 to 400) 1941-1960.

Overall, these patterns closely resemble the global patterns of surface air temperature (Supplementary Figure 2 a, b, c). We can identify the dominant influences on large-scale temperature patterns such as mountain regions (*e.g*. Himalaya and Andes), which are marked by high HDD values, and subtropical desert regions (*e.g.* Sahara and Arabian Peninsula), which are marked by high CDD values.

The global spatial patterns of HDD and CDD are similar for the three studied time periods (1941-1960, 1981-2000, and 2021-2040), but the class boundaries are slightly shifted poleward following the global temperature increase with time (Supplementary Figure 1).

There is a stronger temperature increase from the 1981-2000 average to the 2021-2040 average than from 1941-1960 to 1981-2000 (Supplementary Figure 2 d, e). Accordingly, the HDD and CDD changes become stronger between the two later periods as well (Fig. 1 in the main article).

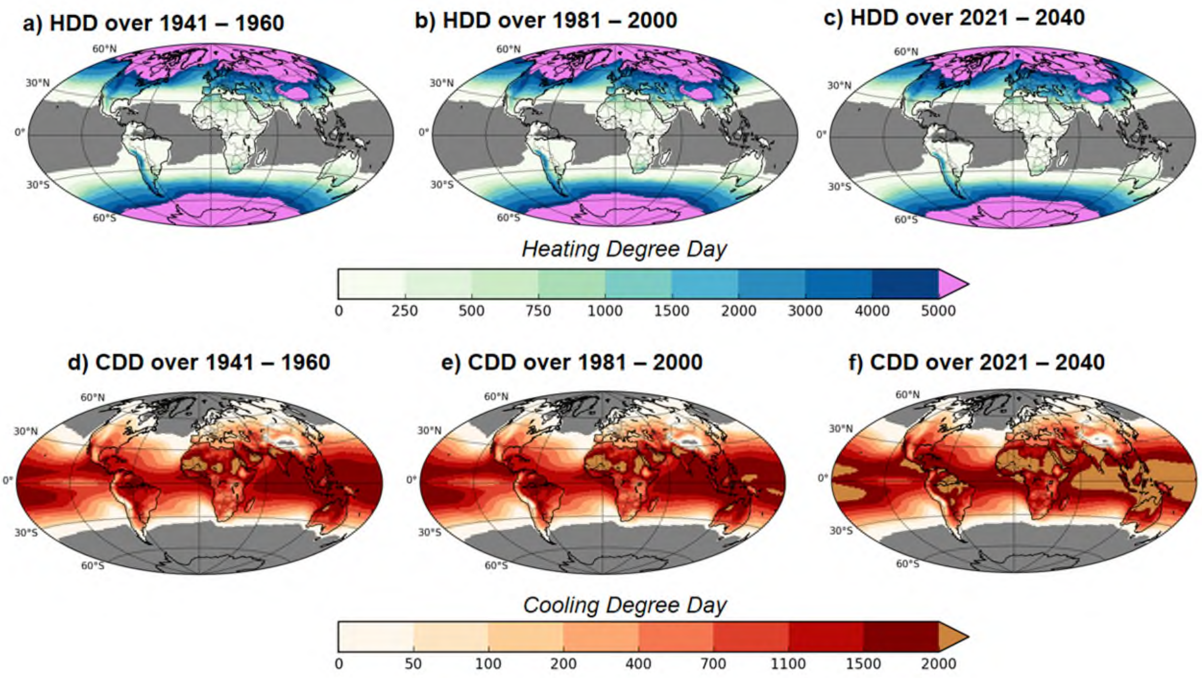

Supplementary Figure 1: Global patterns of annual (top) Heating Degree-Days (HDD) and (bottom) Cooling Degree-Days (CDD). *Annual sums of multi-model HDD and CDD were calculated using the daily mean, minimum and maximum temperatures from 30 CMIP5 models and averaged over the periods (**a**, **d**) 1941-1960, (**b**, **e**) 1981-2000 and (**c**, **f**) 2021-2040. Projections are based on RCP8.5. Areas with an HDD or CDD equal to zero are colored in gray.*

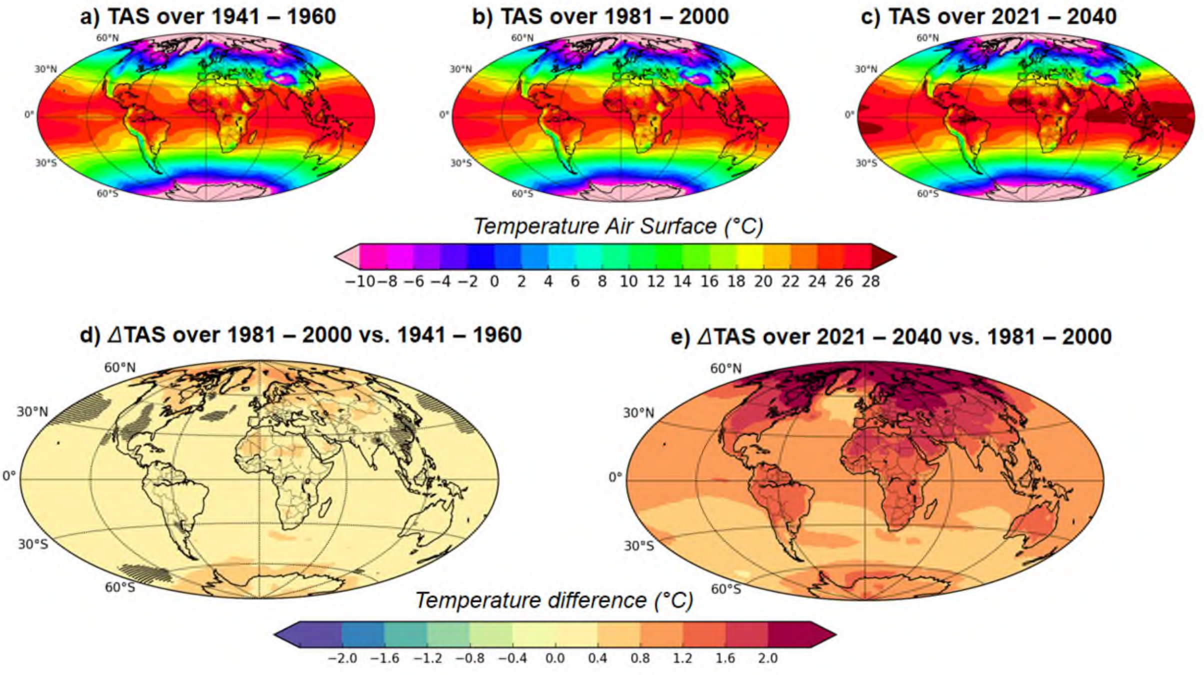

Supplementary Figure 2: Global patterns of Surface Air Temperature (TAS) changes from a multi-model mean of 30 CMIP5 simulations. *(**a**, **b**, **c**) Average TAS for the periods 1941-1960, 1981-2000, and 2021-2040 respectively. (**d**, **e**) Differences in the average temperature (ΔTAS) between the 20-year periods. The areas shaded in gray indicate locations where this difference is not significantly different to zero according to the Student's t test at the 95% confidence level. Projections are based on RCP8.5.*

II. Influence of the Representative Concentration Pathway on future HDD and CDD

The Representative Concentration Pathways (RCP) determine the magnitude of future temperature increase simulated by the CMIP5 models[1]. The results presented in the main article are based on temperature simulations using RCP8.5, and are used in this Supplementary Information as a reference (Supplementary Figure 3) for comparisons.

Projected HDD and CDD changes in the near future (2021-2040) relative to 1981-2000 differ only slightly when comparing the trends simulated with RCP8.5 (the reference) to those simulated with RCP4.5 (comparing Supplementary Figure 3 against Supplementary Figure 4; Supplementary Table 1). For all selected (mega-)city locations, the maximum difference in projected HDD change between the RCPs is 5%; the difference in projected CDD change is below 10% for all but three locations (Supplementary Table 1).

In the far future (2081-2100), temperature projections based on RCP4.5 and RCP8.5 diverge further. Consequently, this leads to a larger difference in the associated changes in HDD and CDD (comparing Supplementary Figure 5 against Supplementary Figure 6).

The reduction in HDD and the increase in CDD on a global scale, which has been observed in the past and is predicted for the near future, continues until the end of the 21st century, in agreement with the projected temperature increase. Although the global HDD and CDD trends in the far future are clear, the inter-model variability remains as large as for the near future period (comparing Supplementary Figure 3 against Supplementary Figure 5).

Supplementary Table 1: Relative changes (in %) in annual Heating Degree-Days (HDD) and Cooling Degree-Days (CDD) for selected cities between the 1981-2000 and 1941-1960 averages, and between the 2021-2040 and 1981-2000 averages. *The values refer to the model grid cells containing the cities. Annual sums of multi-model HDD and CDD were calculated using the daily mean, minimum and maximum temperatures from 30 CMIP5 models, and averaged over the 20-year periods. Projections are based on RCP4.5 and RCP8.5. NS means not significant. The asterisk (*) denotes megacities according to the United Nations definition[2].*

**Location**			**HDD (%)**			**CDD (%)**		
**City**	*Lon (°)*	*Lat (°)*	**1981-2000 -- 1941-1960**	**RCP4.5 1981-2000 – 2021-2040**	**RCP8.5 1981-2000 – 2021-2040**	**1981-2000 – 1941-1960**	**RCP4.5 1981-2000 – 2021-2040**	**RCP8.5 1981-2000 – 2021-2040**
Europe								
**Moscow* (Russia)**	*37.6*	*55.8*	**-4**	**-12**	**-13**	**7**	**59**	**75**
**Berlin (Germany)**	*13.4*	*52.5*	**-4**	**-13**	**-14**	**8**	**73**	**81**
**London* (United Kingdom)**	*-0.1*	*51.5*	**-4**	**-14**	**-14**	**23**	**119**	**125**
**Paris* (France)**	*2.3*	*48.9*	**-4**	**-13**	**-15**	**12**	**76**	**77**
**Rome (Italy)**	*12.5*	*41.9*	**-4**	**-23**	**-25**	**10**	**69**	**76**
**Madrid (Spain)**	*-3.7*	*40.4*	**-3**	**-14**	**-16**	**8**	**60**	**62**
America								
**Montreal (Canada)**	*-73.6*	*45.5*	**-3**	**-12**	**-12**	**14**	**71**	**75**
**New York* (USA)**	*-73.9*	*40.7*	**-4**	**-17**	**-17**	**12**	**65**	**73**
**Los Angeles* (USA)**	*-118.4*	*34.1*	**-4**	**-20**	**-22**	**5**	**27**	**29**
**Mexico City* (Mexico)**	*-99.1*	*19.4*	**-6**	**-24**	**-26**	**10**	**45**	**51**
**São Paulo* (Brazil)**	*-46.6*	*-23.5*	**-11**	**-35**	**-39**	**6**	**32**	**37**
**Buenos Aires (Argentina)**	*-58.4*	*-34.6*	**-5**	**-13**	**-16**	***NS***	**18**	**20**
Asia								
**Beijing* (China)**	*116.4*	*39.9*	**-2**	**-9**	**-10**	***NS***	**31**	**36**
**Seoul* (South Korea)**	*127.0*	*37.6*	**-2**	**-10**	**-12**	***NS***	**50**	**58**
**Tokyo* (Japan)**	*139.8*	*35.7*	**-3**	**-12**	**-14**	**5**	**47**	**53**
**Shanghai* (China)**	*121.5*	*31.2*	***NS***	**-14**	**-16**	***NS***	**33**	**39**
**Delhi* (India)**	*77.2*	*28.7*	**-4**	**-22**	**-24**	**2**	**13**	**14**
**Guangzhou* (China)**	*113.3*	*23.1*	***NS***	**-16**	**-18**	***NS***	**27**	**29**
**Mumbai* (India)**	*72.8*	*19.0*	**-14**	**-42**	**-46**	**4**	**20**	**23**
**Bangkok***	*100.5*	*13.7*	**-7**	**-23**	**-31**	**5**	**20**	**22**

**(Thailand)**								
**Jakarta* (Indonesia)**	*106.8*	*-6.2*	***NS***	**NS**	**NS**	**5**	**18**	**21**
				Africa				
**Cairo* (Egypt)**	*31.3*	*30.1*	**-6**	**-27**	**-31**	**4**	**28**	**32**
**Dakar (Senegal)**	*-17.5*	*14.7*	***NS***	**NS**	**NS**	**4**	**20**	**22**
**Lagos* (Nigeria)**	*3.4*	*6.5*	***NS***	**NS**	**NS**	**2**	**19**	**22**
**Kinshasa* (Congo)**	*15.3*	*-4.3*	***NS***	**-47**	**-47**	**6**	**26**	**30**
**Cape Town (South Africa)**	*18.5*	*-33.9*	**-10**	**-25**	**-28**	**8**	**28**	**32**
				Oceania				
**Perth (Australia)**	*115.8*	*-32.0*	**-9**	**-23**	**-27**	**5**	**30**	**34**
**Melbourne (Australia)**	*145.0*	*-37.8*	**-4**	**-14**	**-17**	***NS***	**31**	**33**

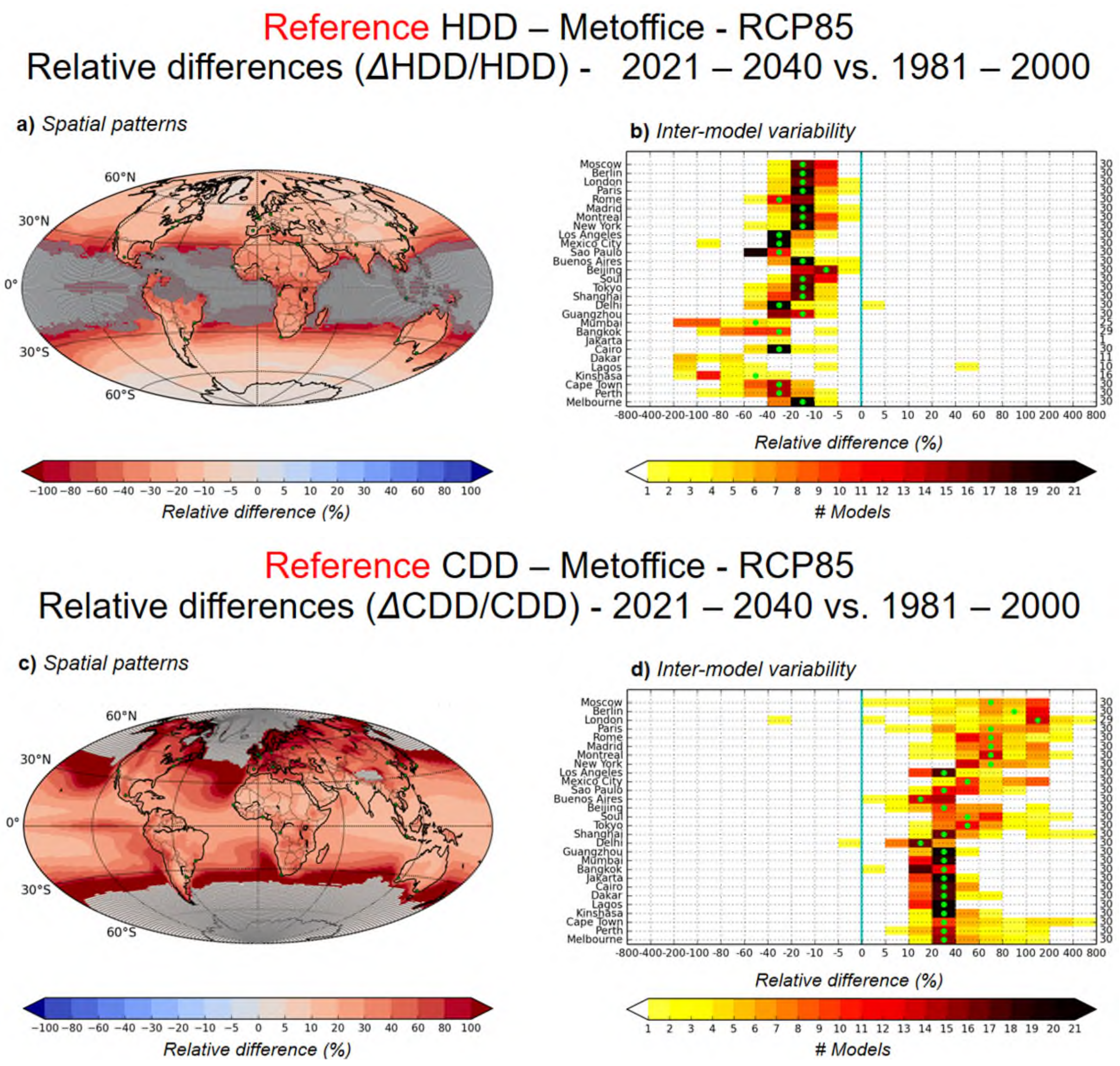

Supplementary Figure 3: Relative differences in Heating Degree-Days (HDD) and Cooling Degree-Days (CDD) between the periods 2021-2040 and 1981-2000. *Annual sums of HDD and CDD are calculated with the Met Office calculation based on daily mean, minimum and maximum temperature from 30 CMIP5 simulations, and averaged over the 20-year periods. All models are interpolated on a 1° x 1° grid. Projections are based on RCP8.5. Differences are given in % compared to the older period. (**a**, **c**) Global patterns for the multi-model mean HDD and CDD. Dark green dots indicate the locations of the cities listed in panels b and d and in Supplementary Table 1. (**b**, **d**) Relative differences in the grid cells of selected cities for individual models. The color bar denotes the number (#) of models in each range of relative difference. The multi-model mean falls in the range marked by the light green dots. Numbers on the right indicate the number of models for which the difference between the two periods is significant. The vertical blue line marks zero.* ***Note: the panels of this figure are also shown in the main article and serve as a reference for comparison with the following figures in this Supplementary Information.***

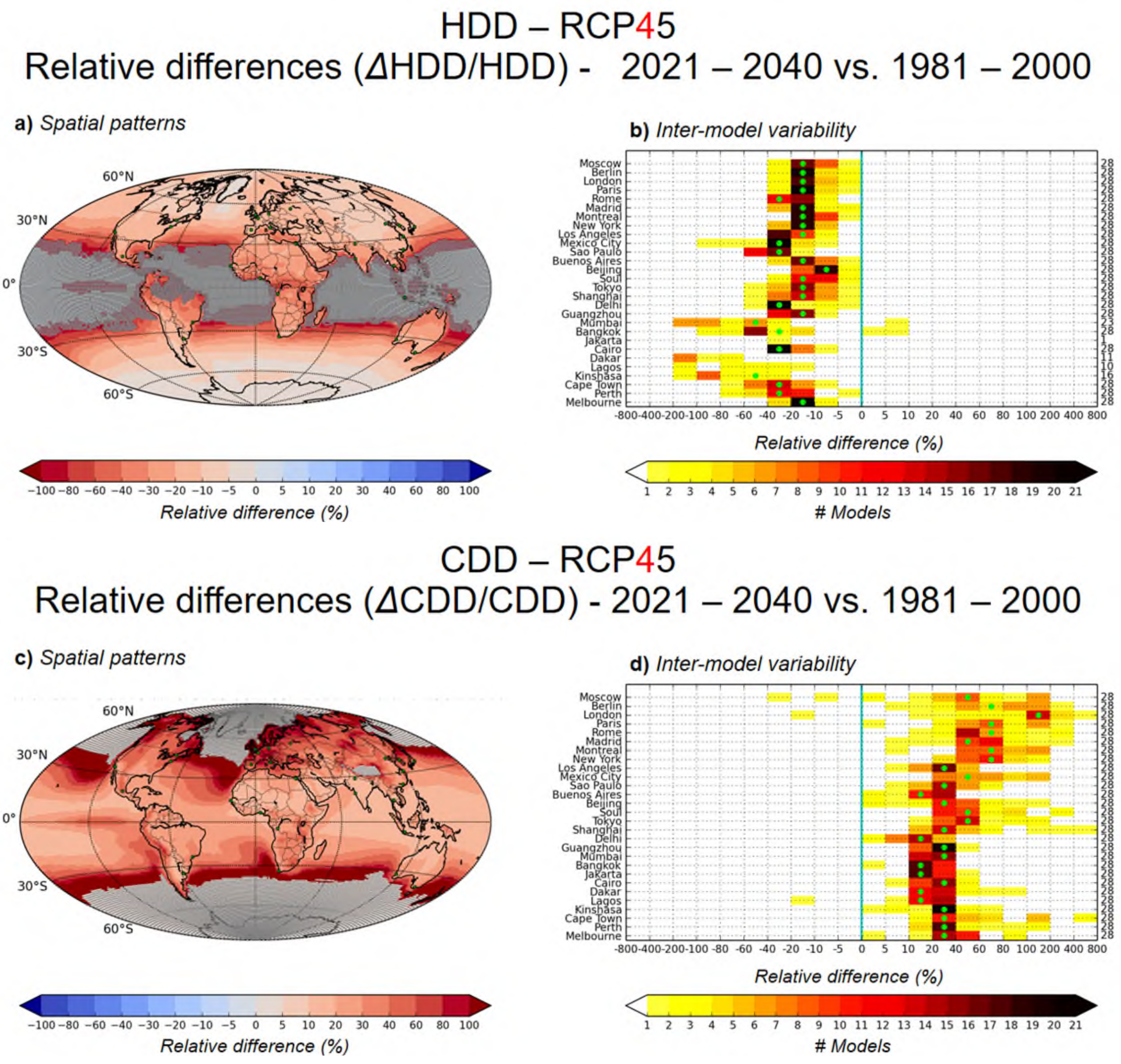

Supplementary Figure 4: *Relative differences in Heating Degree-Days (HDD) and Cooling Degree-Days (CDD) between the periods 2021-2040 and 1981-2000. Annual sums of HDD and CDD are calculated with the Met Office calculation based on daily mean, minimum and maximum temperature from 30 CMIP5 simulations, and averaged over the 20-year periods. All models are interpolated on a 1° x 1° grid. Projections are based on* ***RCP4.5****. Differences are given in % compared to the older period. (****a****,* ***c****) Global patterns for the multi-model mean HDD and CDD. Dark green dots indicate the locations of the cities listed in panels b and d and in Supplementary Table 1. (****b****,* ***d****) Relative differences in the grid cells of selected cities for individual models. The color bar denotes the number (#) of models in each range of relative difference. The multi-model mean falls in the range marked by the light green dots. Numbers on the right indicate the number of models for which the difference between the two periods is significant. The vertical blue line marks zero.*

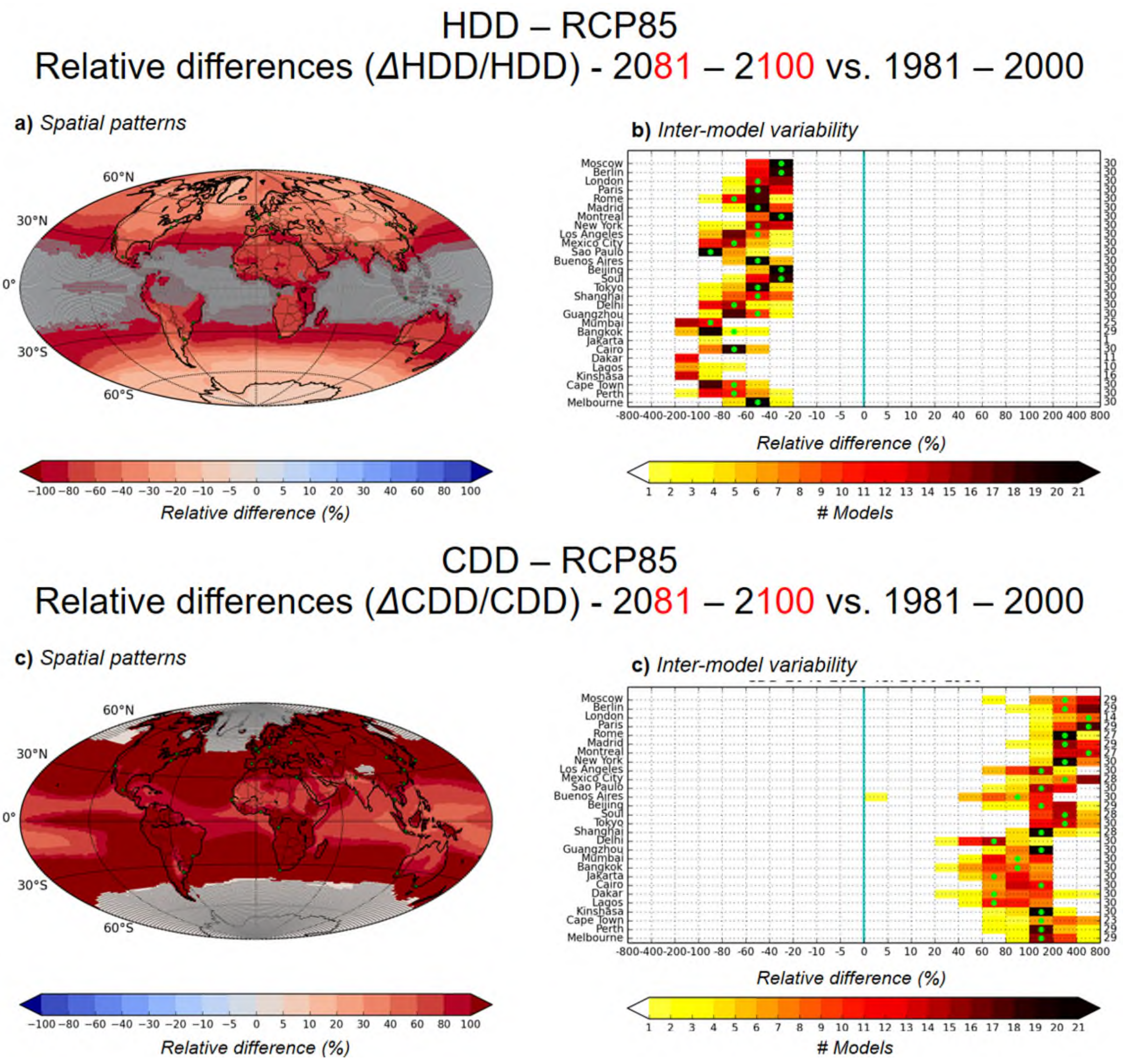

Supplementary Figure 5: *Relative differences in Heating Degree-Days (HDD) and Cooling Degree-Days (CDD) between the periods* ***2081-2100*** *and 1981-2000. Annual sums of HDD and CDD are calculated with the Met Office calculation based on daily mean, minimum and maximum temperature from 30 CMIP5 simulations, and averaged over the 20-year periods. All models are interpolated on a 1° x 1° grid. Projections are based on RCP8.5. Differences are given in % compared to the older period. (****a****,* ***c****) Global patterns for the multi-model mean HDD and CDD. Dark green dots indicate the locations of the cities listed in panels b and d and in Supplementary Table 1. (****b****,* ***d****) Relative differences in the grid cells of selected cities for individual models. The color bar denotes the number (#) of models in each range of relative difference. The multi-model mean falls in the range marked by the light green dots. Numbers on the right indicate the number of models for which the difference between the two periods is significant. The vertical blue line marks zero.*

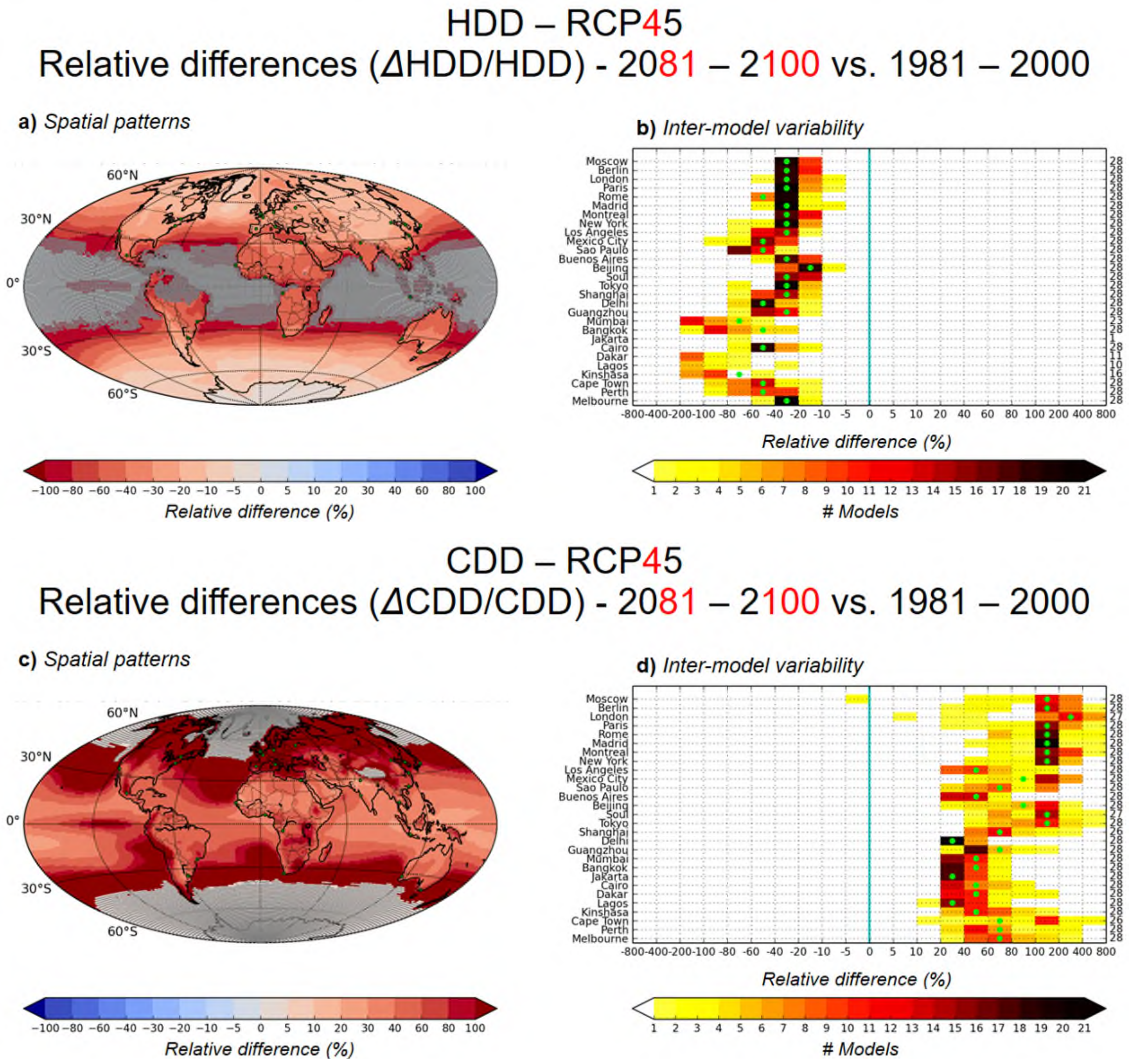

Supplementary Figure 6: *Relative differences in Heating Degree-Days (HDD) and Cooling Degree-Days (CDD) between the periods* ***2081-2100*** *and 1981-2000. Annual sums of HDD and CDD are calculated with the Met Office calculation based on daily mean, minimum and maximum temperature from 30 CMIP5 simulations, and averaged over the 20-year periods. All models are interpolated on a 1° x 1° grid. Projections are based on* ***RCP4.5****. Differences are given in % compared to the older period. (****a****,* ***c****) Global patterns for the multi-model mean HDD and CDD. Dark green dots indicate the locations of the cities listed in panels b and d and in Supplementary Table 1. (****b****,* ***d****) Relative differences in the grid cells of selected cities for individual models. The color bar denotes the number (#) of models in each range of relative difference. The multi-model mean falls in the range marked by the light green dots. Numbers on the right indicate the number of models for which the difference between the two periods is significant. The vertical blue line marks zero.*

III. Alternative steps in the methodology – influence on the results

The different steps in the methodology outlined in the Methods section (main article) require choices that may have an influence on the results. We therefore assessed the robustness of the results presented in the main article by testing in how far they are influenced by the following changes:

a) Different calculation methods are applied to calculate HDD and CDD;

b) The spatial resolution of the multi-model grid is decreased to 2x2°;

c) The length of the time periods over which HDD and CDD are averaged is increased to 30 years;

d) It is tested whether the inter-model variability is reduced when biases in simulated temperatures are corrected based on observations before calculating HDD and CDD;

e) The multi-model HDD and CDD obtained by averaging HDD and CDD calculated for each individual model are compared to HDD and CDD calculated from multi-model daily temperatures.

The effects of these changes in the methodology are illustrated in the following subsections for each aspect (a to e) individually, focusing on (1) the relative change in annual HDD and CDD between the 1981-2000 and 2021-2040 averages (corresponding to Fig. 2 b and d in the main article) to show the global patterns; and (2) the inter-model variability for selected megacities in the relative HDD and CDD change between these periods (corresponding to Fig. 3 b and d in the main article) to show the magnitude of change in densely populated regions.

a. Different Degree-Days calculation methods

Why this test? To test if the results are influenced by the Degree-Days calculation method, we compare the two most widely used approaches, the UK Met Office calculation (applied to obtain our results presented in the main article) and a simpler calculation used in the USA.

Method The UK Met Office calculation, based on daily minimum, mean and maximum temperatures with base temperatures of 15.5 °C for heating and 22 °C for cooling, is replaced by the US calculation, which is based only on the daily mean temperature and is applied with a unique base temperature of 18.3 °C for both heating and cooling [3]. For the three studied time periods, with the multi-model mean, the difference between the 20-year averages of HDD (resp. CDD) from the two calculations is computed in each grid cell.

Result The US calculation with a unique base temperature of 18.3 °C leads to consistently higher HDD and CDD values than the UK Met Office calculation (Supplementary Figure 7), because the temperature threshold for heating is higher (18.3 °C vs. 15.5 °C) and the

temperature threshold for cooling is lower (18.3 °C vs. 22 °C). Nevertheless there is a high level of correlation between the two methods, with correlation coefficients of 0.99 for HDD and 0.97 for CDD, when correlating grid cell values for each of the studied time periods. Despite the differences in HDD and CDD values depending on the calculation method, the spatial patterns of the differences between the two calculation methods are constant over time (Supplementary Figure 7). Consequently, the spatial patterns of the relative HDD and CDD changes are not sensitive to the Degree-Days calculation method, and the simulated trends are similar to our reference (comparing Supplementary Figure 8 against Supplementary Figure 3). The inter-model variability is also in a similar range with both calculations.

The question of the calculation method, i.e. whether daily mean temperature, or daily mean, minimum and maximum temperature are an adequate representation of the diurnal temperature cycle, can be overcome if temperature simulations with an output at higher temporal resolution, such as hourly or 3-hourly, are available. These enable the calculation of Degree-Hours based on the modeled diurnal cycle of temperature at the grid-cell scale.

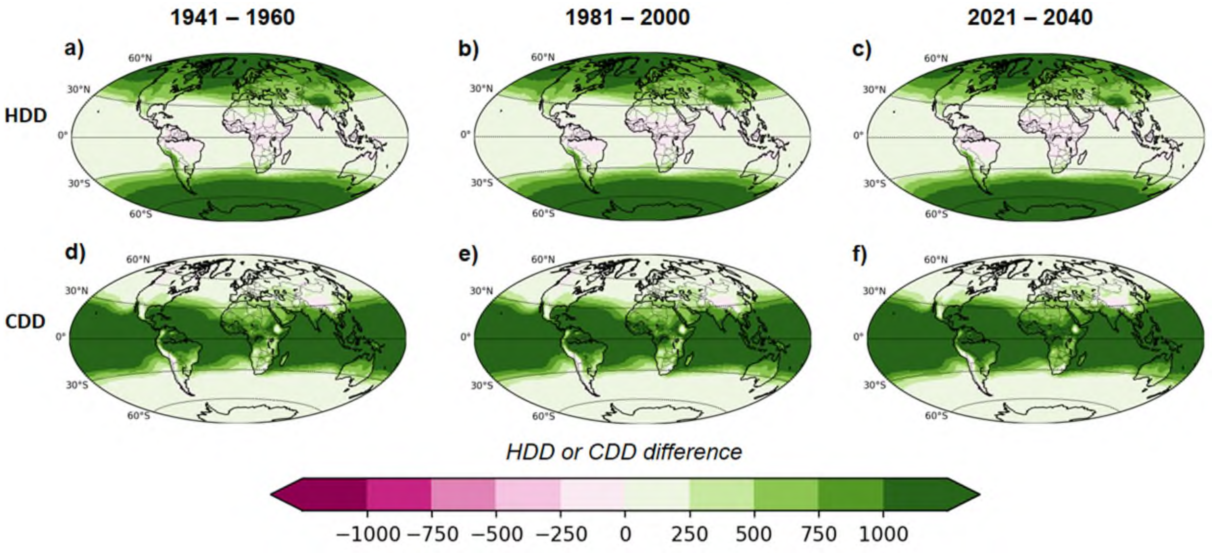

Supplementary Figure 7: Differences of Heating Degree-Days (HDD) resp. Cooling Degree-Days (CDD) calculated with the UK Met Office method and HDD resp. CDD calculated with the US standard method. *The differences are shown for 20-year averages of (**a**, **b**, **c**) annual HDD, and (**d**, **e**, **f**) annual CDD, for the periods 1941-1960, 1981-2000, and 2021-2040.*

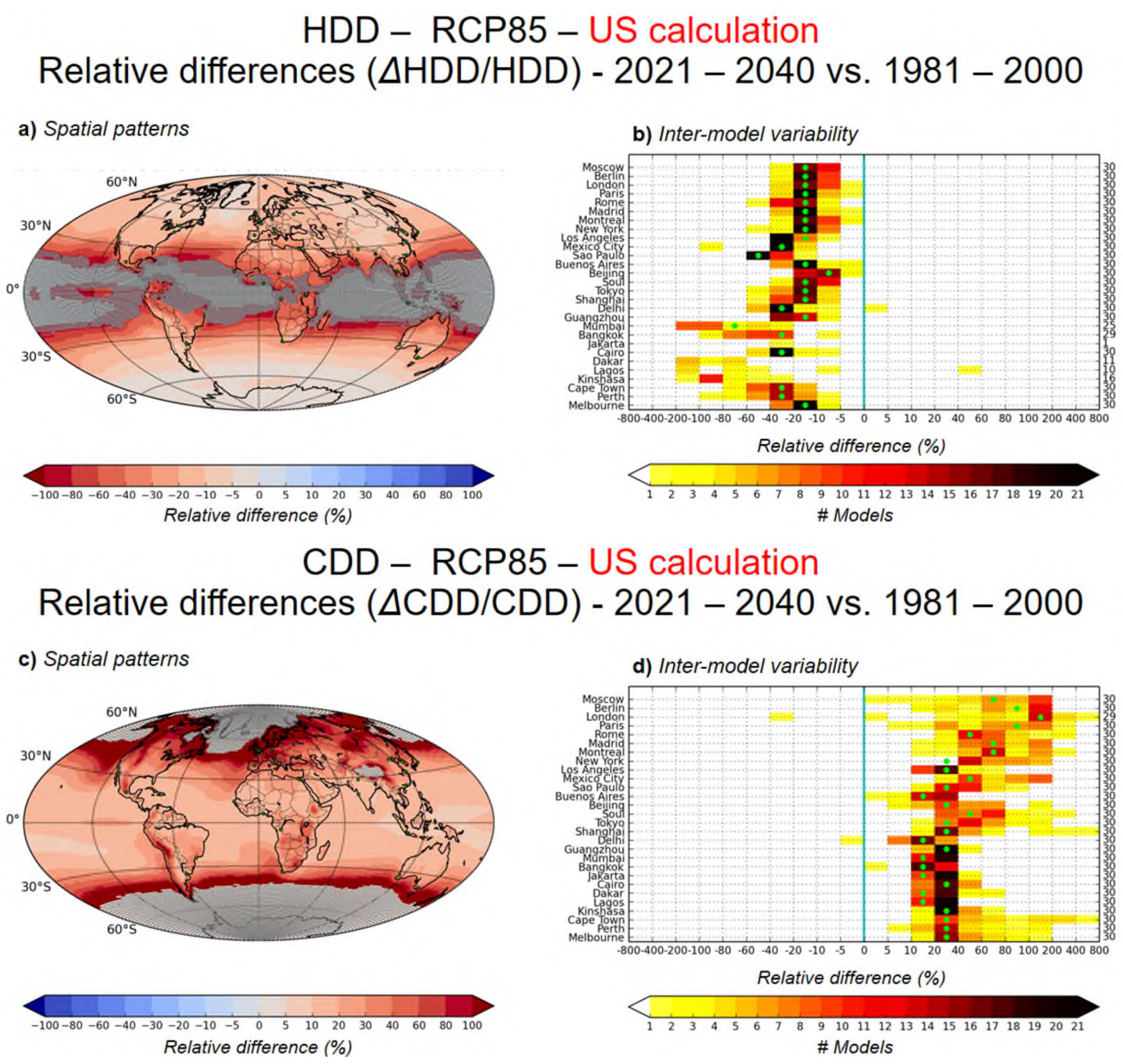

Supplementary Figure 8: *Relative differences in Heating Degree-Days (HDD) and Cooling Degree-Days (CDD) between the periods 2021-2400 and 1981-2000. Annual sums of HDD and CDD are calculated* ***with the US standard method and a unique base temperature of 18.3 °C for both heating and cooling*** *based on daily mean, minimum and maximum temperature from 30 CMIP5 simulations, and averaged over the 20-year periods. All models are interpolated on a 1° x 1° grid. Projections are based on RCP8.5. Differences are given in % compared to the older period. (**a**, **c**) Global patterns for the multi-model mean HDD and CDD. Dark green dots indicate the locations of the cities listed in panels b and d and in Supplementary Table 1. (**b**, **d**) Relative differences in the grid cells of selected cities for individual models. The color bar denotes the number (#) of models in each range of relative difference. The multi-model mean falls in the range marked by the light green dots. Numbers on the right indicate the number of models for which the difference between the two periods is significant. The vertical blue line marks zero.*

b. Interpolating the models on a grid of 2° resolution

Why this test? The native grid resolution of the CMIP5 models used in this study (Supplementary Table 2) varies between 0.7484 and 3.7111 degrees latitude (at the equator) and between 0.75 and 3.75 degrees longitude, with an average resolution of 1.8186 degrees latitude and 2.2438 degrees longitude. This implies that our spatial interpolation on a 1° by 1° grid creates data at a higher spatial resolution than the model output for many of the models.

Method The multi-model mean HDD and CDD were therefore calculated as outlined in the Methods section of the main article, except for step 4. The HDD and CDD 20-year means were instead interpolated spatially on a multi-model grid of 2° by 2°.

Result Despite the coarser resolution, the same spatial patterns of HDD and CDD changes are apparent on a global scale (Supplementary Figure 9 a, c). The magnitude of change for the studied megacities and the inter-model variability also remain very similar (Supplementary Figure 9 b, d).

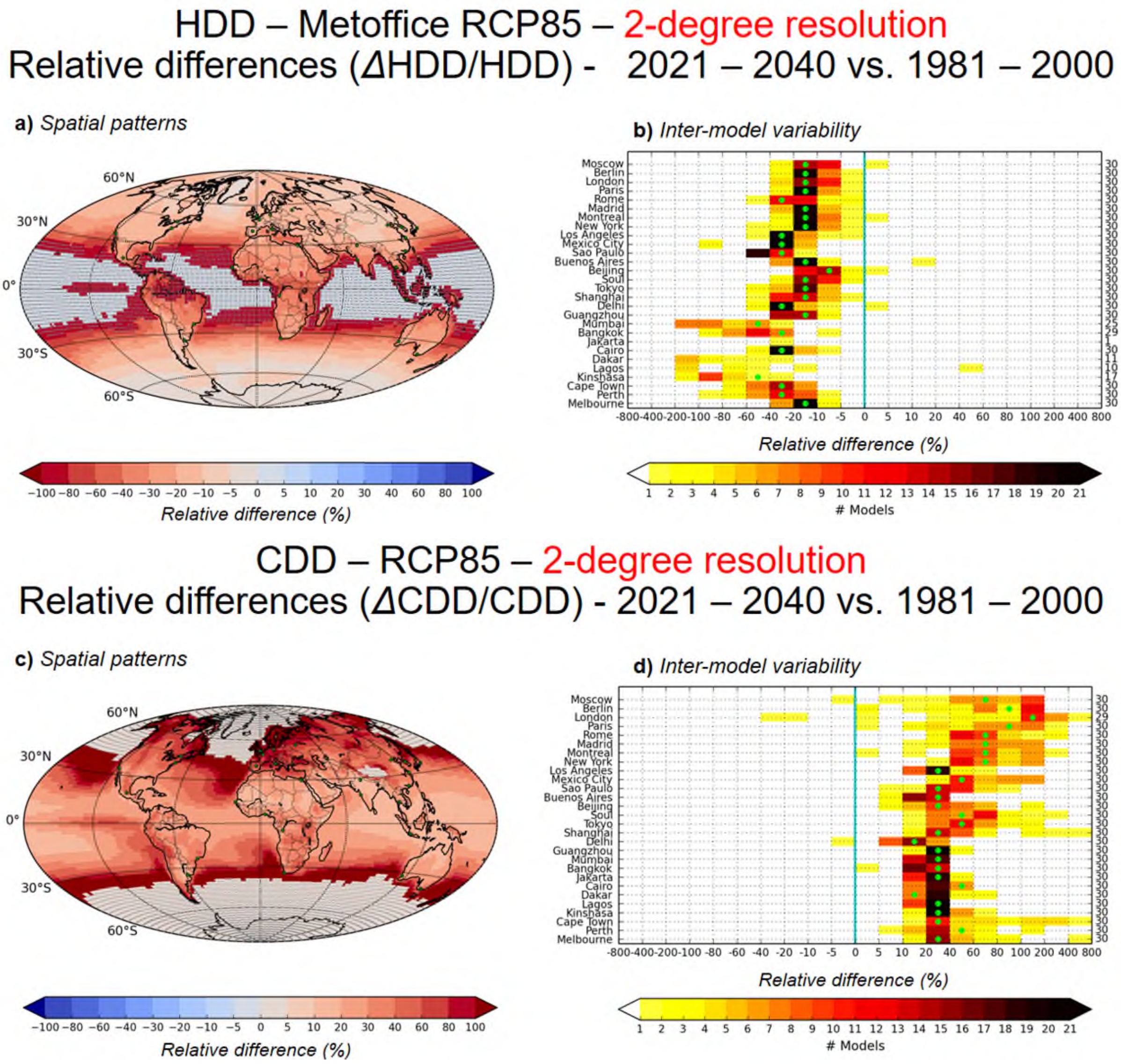

Supplementary Figure 9: *Relative differences in Heating Degree-Days (HDD) and Cooling Degree-Days (CDD) between the periods 2021-2040 and 1981-2000. Annual sums of HDD and CDD are calculated with the Met Office calculation based on daily mean, minimum and maximum temperature from 30 CMIP5 simulations, and averaged over the 20-year periods. All models are interpolated on a* ***2° x 2° grid****. Projections are based on RCP8.5. Differences are given in % compared to the older period. (****a****,* ***c****) Global patterns for the multi-model mean HDD and CDD. Dark green dots indicate the locations of the cities listed in panels b and d and in Supplementary Table 1. (****b****,* ***d****) Relative differences in the grid cells of selected cities for individual models. The color bar denotes the number (#) of models in each range of relative difference. The multi-model mean falls in the range marked by the light green dots. Numbers on the right indicate the number of models for which the difference between the two periods is significant. The vertical blue line marks zero.*

c. Influence of the length of the time spans over which annual HDD and CDD are averaged

Why this test? To illustrate the temporal evolution of HDD and CDD we base our analysis on 20-year averages (as e.g. in IPCC 2013, AR5 WGI, Annex I). This enables a comparison of our results with other investigation of climate change and its impacts in the context of the IPCC Assessment Reports. The World Meteorological Organization uses 30-year temperature averages to define a climatology. It is therefore tested whether the detected changes over time depend on the chosen length of the time span over which HDD and CDD are averaged.

Method The changes in HDD and CDD were calculated for three 30-year time spans, extending our 20-year time spans (1941-1960, 1981-2000, and 2021-2040) by 5 years at the beginning and at the end to 1936-1965, 1976-2005, and 2016-2045.

Result We observe very similar spatial patterns and temporal evolution of HDD and CDD when comparing two time spans of either 20 or 30 years, centered on the same year (Supplementary Figure 10). The long-term trends remain clear, and our conclusions based on the 20-year time spans would not be changed with the longer averaging period.

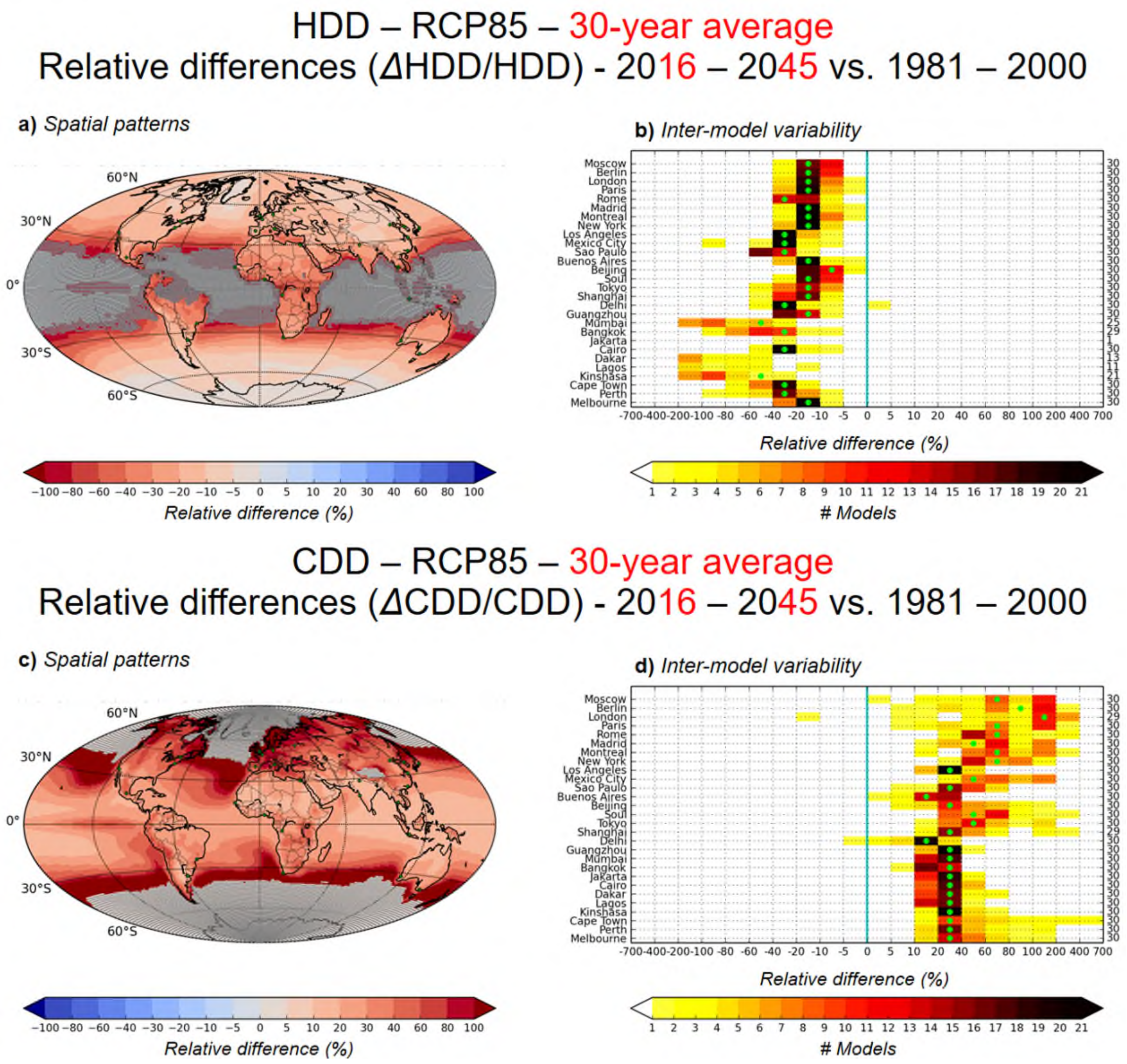

Supplementary Figure 10: *Relative differences in Heating Degree-Days (HDD) and Cooling Degree-Days (CDD) between the periods* ***2016-2045*** *and* ***1976-2005****. Annual sums of HDD and CDD are calculated with the Met Office calculation based on daily mean, minimum and maximum temperature from 30 CMIP5 simulations, and averaged over the* ***30-year periods****. All models are interpolated on a 1° x 1° grid. Projections are based on RCP8.5. Differences are given in % compared to the older period. (****a****,* ***c****) Global patterns for the multi-model mean HDD and CDD. Dark green dots indicate the locations of the cities listed in panels b and d and in Supplementary Table 1. (****b****,* ***d****) Relative differences in the grid cells of selected cities for individual models. The color bar denotes the number (#) of models in each range of relative difference. The multi-model mean falls in the range marked by the light green dots. Numbers on the right indicate the number of models for which the difference between the two periods is significant. The vertical blue line marks zero.*

d. Correcting model biases based on observations

Why this test? The historical temperature simulations of the CMIP5 models are known to have systematic biases compared to observations[4]. It can be assumed that the inter-model variability for HDD and CDD change (as presented in the main article Fig. 3) could be reduced when model biases in simulated temperature are corrected before HDD and CDD calculation.

Method Gridded observational data (interpolated from stations) is only available for land areas. The model biases are corrected based on the CRU TS 4.0 data set at 0.5° resolution[5] following these steps for each of the CMIP5 models:

1. The CRU data is interpolated on the native resolution of the model.
2. Monthly mean temperatures are calculated over the reference period 1981-2000 for the model and the corresponding interpolated CRU data.
3. The model bias in monthly mean temperature is calculated for each grid cell by subtracting the value or the CRU data from the value of the model.
4. This monthly bias in each grid cell is interpolated on 365 values to obtain a daily bias.
5. A bias-corrected daily temperature is calculated for each grid cell by subtracting the daily bias from the daily modeled temperature.
6. Annual HDD and CDD (averaged over the 20-year periods) are calculated using the bias-corrected daily temperature according to the steps outlined in the Methods section (main article).

Result Both positive and negative biases are observed. The biases between models and observations show a large spatial and temporal variability, i.e. for a given model, the biases are not the same for all regions and change throughout the year. The spatio-temporal differences in the bias are illustrated with two models for January (Supplementary Figure 11) and June (Supplementary Figure 12). For example, in the IPSL-CM5A-MR model, the bias in tropical regions changes sign between January and June. In the MPI-ESM-MR model, the bias in Australia and the USA also changes sign. The MIP-ESM-MR model shows a strong positive bias in northern Asia in January, whereas the bias is less strong in June.

This shows the importance of the monthly, grid-scale based bias correction for temperature. However, spatial patterns of HDD and CDD change over land areas are very similar with and without bias correction (Supplementary Figure 13a, c). A reduction in the inter-model variability could be expected. However, this inter-model variability for future HDD and CDD change in the studied megacities is barely reduced by the correction (Supplementary Figure 13b, d). The slight decrease of the variability can be explained by the increasing number of models showing non-significant changes, especially in tropical and subtropical cities in Africa and Asia.

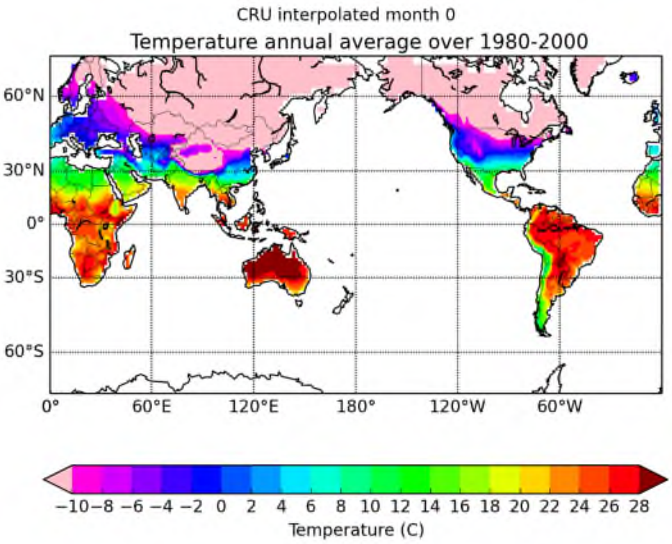

MPI **- January -** **IPSL**

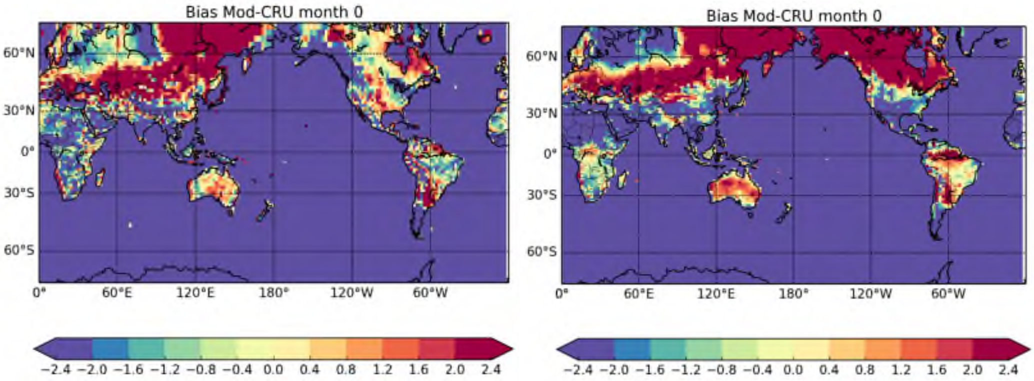

Supplementary Figure 11: *Average January temperature from (**top**) the CRU TS 4.0 data set* [5] *and (**bottom**) the associated bias (in °C) for the MPI-ESM-MR and IPSL-CM5A-MR models. The bias is only calculated for continental surfaces.*

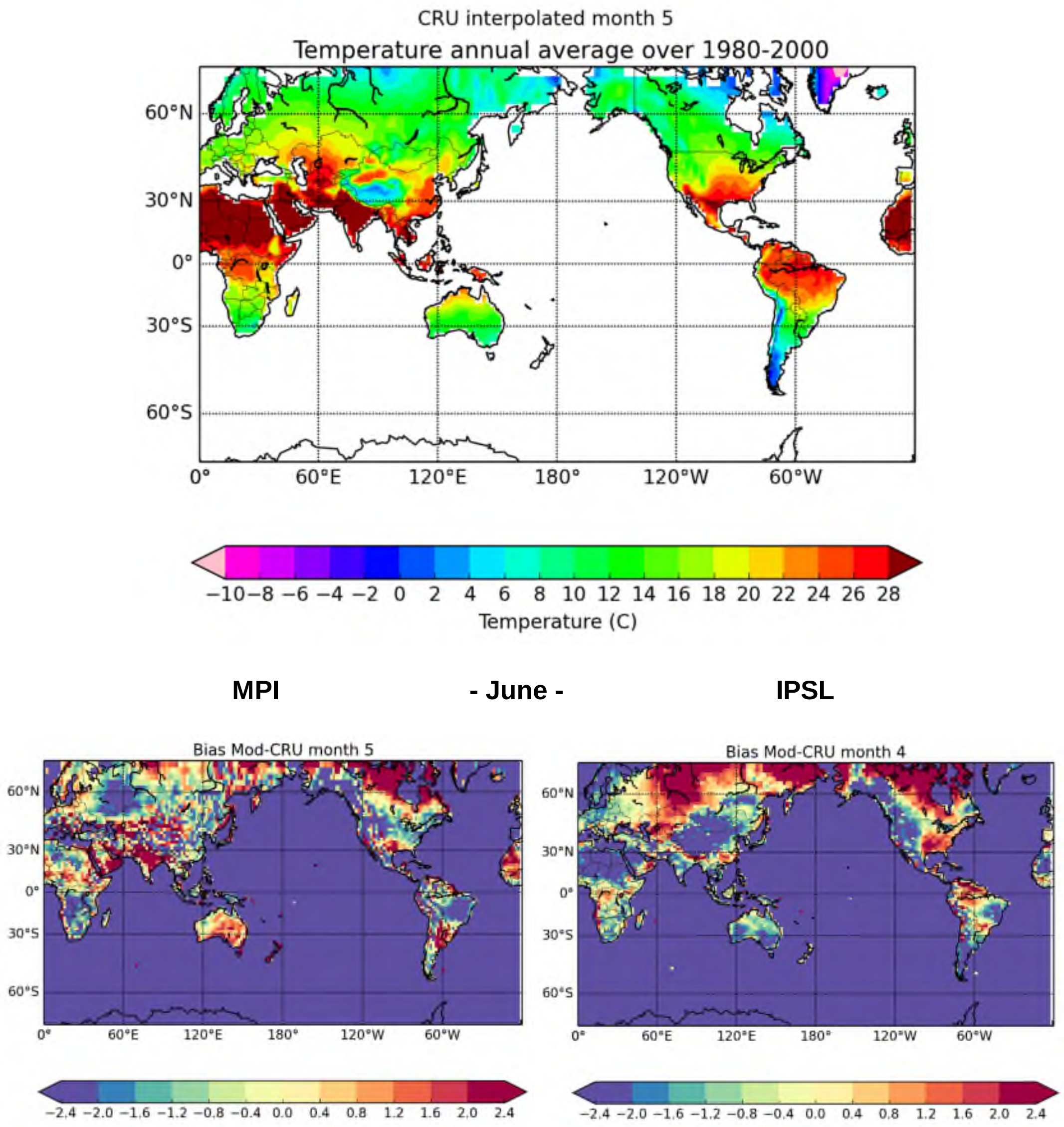

Supplementary Figure 12: *Average June temperature from (**top**) the CRU TS 4.0 data set* [5] *and (**bottom**) the associated bias (in °C) for the MPI-ESM-MR and IPSL-CM5A-MR models. The bias is only calculated for continental surfaces.*

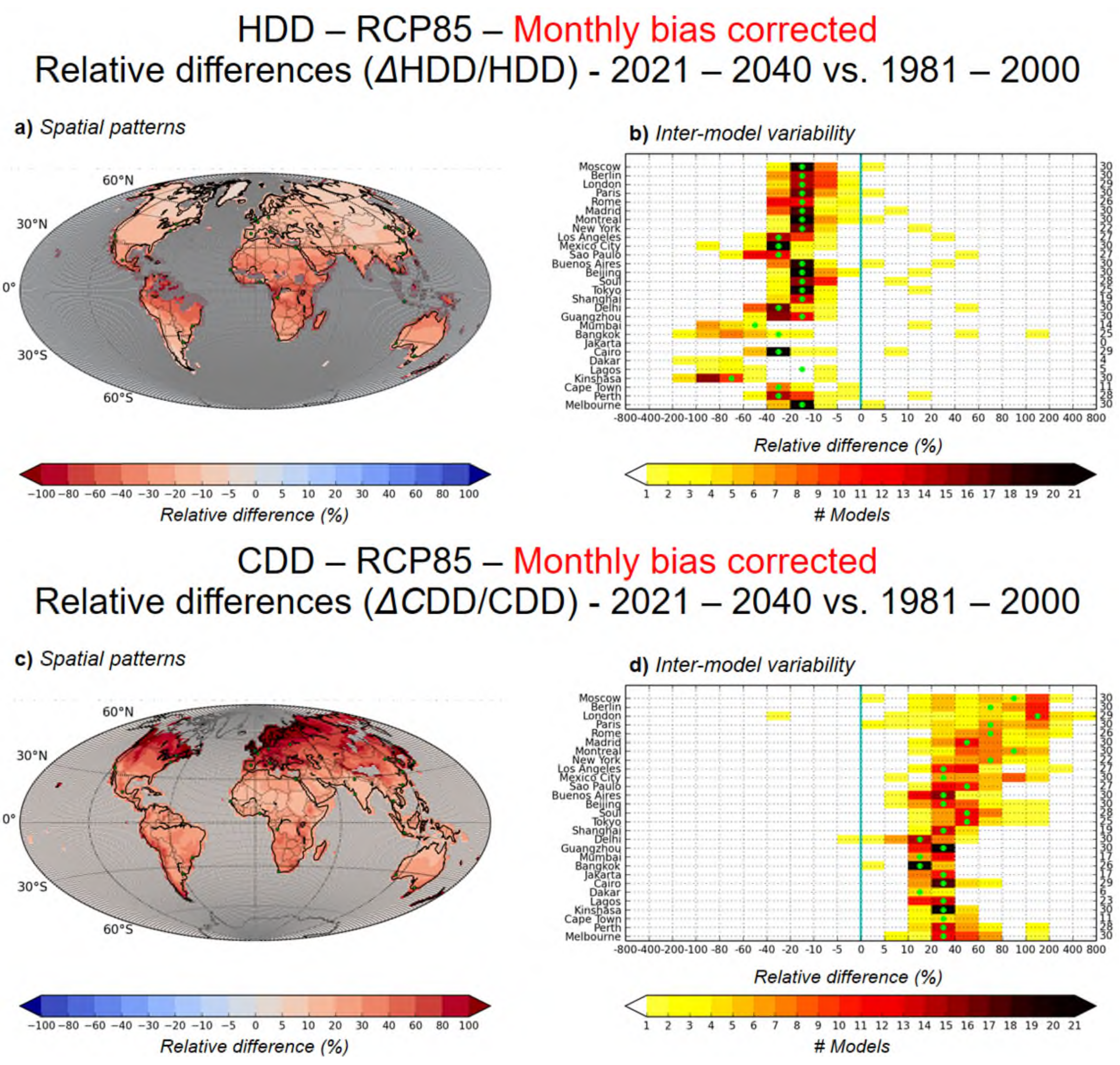

Supplementary Figure 13: *Relative differences in Heating Degree-Days (HDD) and Cooling Degree-Days (CDD) between the periods 2021-2040 and 1981-2000. Annual sums of HDD and CDD are calculated with the Met Office calculation based on daily mean, minimum and maximum temperature from 30 CMIP5 simulations, and averaged over the 20-year periods. All models are interpolated on a 1° x 1° grid. Projections are based on RCP8.5. Differences are given in % compared to the older period.* ***Simulated temperatures are corrected for the bias between the models and monthly mean temperatures for the period 1981-2000 from the CRU TS 4.0 data set (Harris et al. 2014).*** *(**a**, **c**) Global patterns for the multi-model mean HDD and CDD. Dark green dots indicate the locations of the cities listed in panels b and d and in Supplementary Table 1. (**b**, **d**) Relative differences in the grid cells of selected cities for individual models. The color bar denotes the number (#) of models in each range of relative difference. The multi-model mean falls in the range marked by the light green dots. Numbers on the right indicate the number of models for which the difference between the two periods is significant. The vertical blue line marks zero.*

e. Calculating HDD and CDD from multi-model daily temperatures

Why this test? Because HDD and CDD are dependent on thresholds, calculating HDD and CDD for individual models and then calculating a multi-model mean HDD and CDD does not lead to the same result as calculating first a multi-model mean of daily temperatures, and then calculating HDD and CDD from these multi-model temperatures.

Method Instead of following the steps outlined in the Methods section (main article), we first calculate the multi-model means of daily mean, minimum and maximum temperatures, by spatially interpolating daily temperatures of each model on the same grid of 1° by 1°. HDD and CDD are then calculated based on the multi-model daily temperatures.

Result Averaging the simulated temperatures of all models first reduces the temperature variability compared to the temperature variability of all individual models. If the multi-model mean temperature of a given day and a given grid cell does not cross a threshold, this would result in zero HDD or CDD. However, some of the individual models may produce a temperature below/above the threshold for this day and grid cell. In that case, these models would yield HDD or CDD > 0, and the multi-model mean CDD would also be above 0. Consequently, the method tested here leads to lower HDD and CDD because there are fewer temperature extremes in the multi-model temperature compared to all individual models.

Nevertheless, the global patterns of change remain similar with both calculation methods (Supplementary Figure 14). These patterns are therefore not dependent on extreme temperature events, which could be averaged out in the multi-model daily temperature. Rather, they reflect the average climatology, which is similar for both methods.

Still, it is advised to work with temperature simulations of all available climate models rather than multi-model temperatures to provide a range of possible future temperatures for calculating Degree-Days in the context of energy demand predictions. Although this methodology leads to similar simulated trends in the heating and cooling proxies, it hides the uncertainties due to the large inter-model variability.

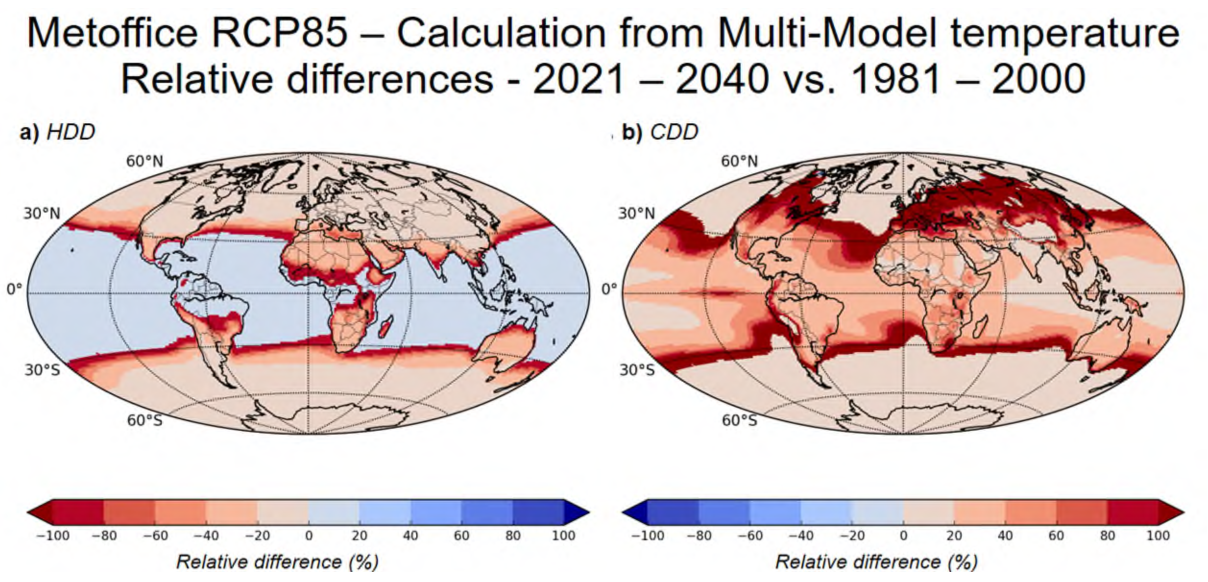

Supplementary Figure 14: *Relative differences in Heating Degree-Days (HDD) and Cooling Degree-Days (CDD) between the periods 2021-2040 and 1981-2000. Annual sums of HDD and CDD are calculated with the Met Office calculation based on a* ***multi-model mean of daily mean, minimum and maximum temperature*** *from 30 CMIP5 simulations, and averaged over the 20-year periods. All models are interpolated on a 1° x 1° grid. Projections are based on RCP8.5. Differences are given in % compared to the older period.*

IV. Sensitivity of HDD and CDD trends to the choice of base temperature

In this section, we investigate the influence of the base temperature (Tbase) on the results, i.e. on the trends in HDD and CDD. Different base temperatures are applied in different countries (see Methods section in the main article). With a different Tbase, the annual sum of HDD or CDD changes. For example, with a higher Tbase the annual sum of CDD will be lower. However, if the difference caused by Tbase is constant in time, the HDD and CDD trends that we show using a specific Tbase will be similar.

Method The 20-year averages of HDD and CDD annual sums are calculated for the three studied periods, using the UK Met Office calculation as explained in the Methods section (main article), but using two different base temperatures: 15.5 and 18.3 °C for HDD and 22 and 18.3 °C for CDD. Correlation coefficients between HDD (resp. CDD) using different Tbase are calculated for each time period. Then, for each grid cell, the differences between HDD (resp. CDD) using the different Tbase are calculated for each time period.

Result The correlation coefficients of the HDD and CDD calculated with the two base temperatures are the same for the three periods (R = 0.99 for HDD and R = 0.93 for CDD). The spatial patterns of the differences are constant in time both for HDD and for CDD, i.e. they are the same for the three periods studied (Supplementary Figure 15). In conclusion, the differences caused by the choice of the base temperature do not modify the climate-driven energy demand trends that we infer from HDD and CDD as heating and cooling proxies.

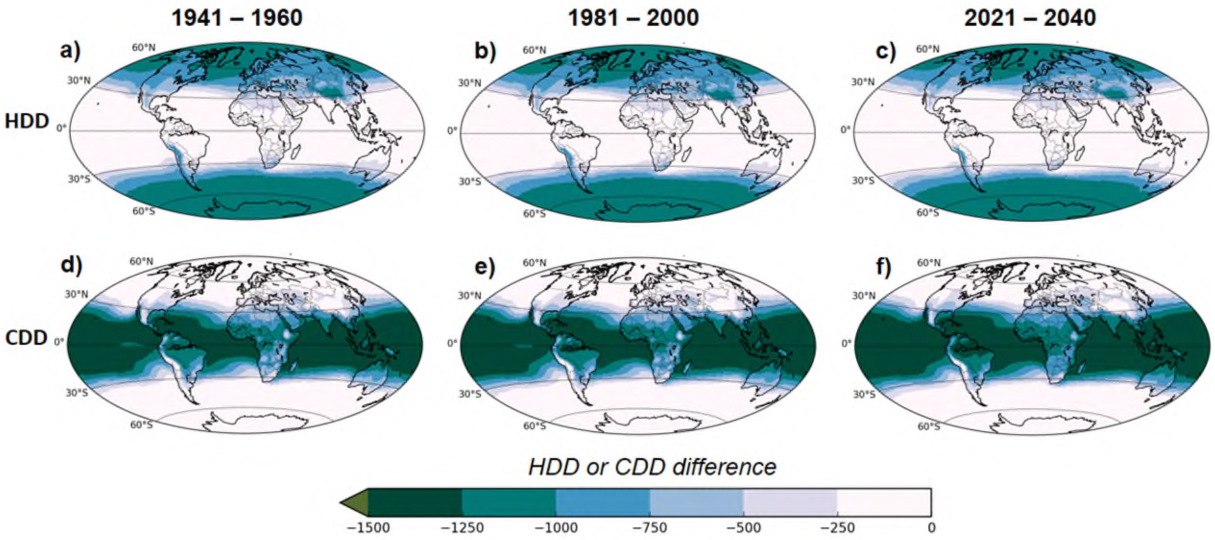

Supplementary Figure 15: Differences of HDD calculated with a base temperature (Tbase) of 15.5 °C and HDD calculated with Tbase of 18.3 °C and differences of CDD calculated with Tbase of 18.3 °C and CDD calculated with Tbase of 22 °C. *The differences are shown for 20-year averages of (**a**, **b**, **c**) annual HDD, and (**d**, **e**, **f**) CDD, for the periods 1941-1960, 1981-2000, and 2021-2040.*

V. CMIP5 models

Heating and Cooling Degree-Days are calculated in this study using historical simulations and temperature projections from 30 CMIP 5 models[6], listed in Supplementary Table 2.

The HadGEM2-CC and HadGEM2-ES models use 30 days for each month, i.e. 360 values in one year instead of 365, or 1.4% less days than the other models. This could lead to an underestimation of HDD and CDD as these variables are summed (and not averaged) over one year. However, the HDD and CDD calculated from temperatures simulated by these models fall in the range of HDD and CDD from the other models. The BNU-ESM model simulation starts in 1950, so the earliest studied time period covers only 1950-1960 instead of 1941-1960.

Supplementary Table 2: List of the 30 models from the Coupled Model Intercomparison Project phase 5 – CMIP5 [6] used in this study, and corresponding institutions.

	**Model name**	**Institution**	**Institution abbreviation**
1	bcc-csm1-1-m	Beijing Climate Center, China Meteorological Administration	BCC
2	BNU-ESM	Beijing Normal University (China)	BNU
3	CanESM2	Canadian Centre for Climate Modelling and Analysis	CCCma
4	CMCC-CESM	Centro Euro-Mediterraneo per I Cambiamenti Climatici (Italy)	CMCC
5	CMCC-CM		
6	CMCC-CMS		
7	CNRM-CM5	Centre National de Recherches Météorologiques / Centre Européen de Recherche et Formation Avancée en Calcul Scientifique (France)	CNRM-CERFACS
8	ACCESS1-0	Commonwealth Scientific and Industrial Research Organization / Bureau of Meteorology (Australia)	CSIRO-BOM
9	ACCESS1-3		
10	inmcm4	Institute for Numerical Mathematics (Russia)	INM
11	IPSL-CM5A-LR	Institut Pierre-Simon Laplace (France)	IPSL
12	IPSL-CM5A-MR		
13	IPSL-CM5B-LR		
14	MIROC-ESM	Japan Agency for Marine-Earth Science and Technology, Atmosphere and Ocean Research Institute (The University of Tokyo), and National Institute for Environmental Studies	MIROC
15	MIROC-ESM-CHEM		
16	MIROC5		
17	HadGEM2-CC	Met Office Hadley Centre (United Kingdom)	MOHC
18	HadGEM2-ES		
19	MPI-ESM-LR	Max Planck Institute for Meteorology (Germany)	MPI-M
20	MPI-ESM-MR		
21	MRI-CGCM3	Meteorological Research Institute (Japan)	MRI
22	MRI-ESM1		
23	CCSM4	National Center for Atmospheric Research (USA)	NCAR
24	NorESM1-M	Norwegian Climate Centre	NCC
25	HadGEM2-AO	National Institute of Meteorological Research (South Korea)	NIMR
26	GFDL-CM3	National Oceanic and Atmospheric Administration / Geophysical Fluid Dynamics Laboratory (USA)	NOAA-GFDL
27	GFDL-ESM2G		
28	GFDL-ESM2M		
29	CESM1-BGC	National Science Foundation / National Center for Atmospheric Research (USA)	NSF-NCAR
30	CESM1-CAM5		

VI. References

1. van Vuuren, D. P. *et al.* The representative concentration pathways: An overview. *Clim. Change* **109**, 5–31 (2011).
2. *Population Prospects: The 2015 Revision*. (2015).
3. Day, T. *Degree-days: theory and application*. *Technical memoranda (Chartered Institution of Building Services Engineers)* (2006).
4. Christensen, J. H. & Boberg, F. Temperature dependent climate projection deficiencies in CMIP5 models. *Geophys. Res. Lett.* **39**, 2012GL053650 (2012).
5. Harris, I., Jones, P. D., Osborn, T. J. & Lister, D. H. Updated high-resolution grids of monthly climatic observations - the CRU TS3.10 Dataset. *Int. J. Climatol.* **34**, 623–642 (2014).
6. Taylor, K. E., Stouffer, R. J. & Meehl, G. a. An Overview of CMIP5 and the Experiment Design. *Bull. Am. Meteorol. Soc.* **93**, 485–498 (2012).
