# Supplementary material for: Large uncertainties in trends of energy demand for heating and cooling under climate change
Source: Nat Commun. 2021 Aug 31;12:5197. doi: 10.1038/s41467-021-25504-8 (PMC8408235; doi:10.1038/s41467-021-25504-8)
Supplement: Supplementary file 2 — Peer Review File [file 41467_2021_25504_MOESM2_ESM.pdf]

Peer Review File

Reviewer comments, first round –

Reviewer #1 (Remarks to the Author):

The paper presents an analysis of how the uncertainties of climate models affect the energy demand projections globally in a warming world. Authors analyzes various scenarios and reports the results on heating and cooling proxies, global patterns in heating and cooling, trends in energy demands, inter-model variability, by the end of the century energy demands and robustness of the trends. The paper is well written, but I have major concerns regarding some of the basic concepts.

1. In page 2 of the Supplement, (Pg. 2, line 38-39), the authors conclude that the relative change in the heat flux (F) between the inside and the outside of a building is equivalent to the relative change in temperature. However, this change in heat flux is independent of the building's properties only when the building characteristics don't change over time. This assumption is a strong one, especially when long-term energy demand forecast is referred to. For example, the efficiency of buildings is changing fast along the increase in efficiency of the equipment such as air conditioner or heaters. Moreover, there is also a shift in the type of energy (for example, electricity, natural gas, renewables) being used for space cooling or heating. Thus, this is a very important context and I want to see how the authors are accounting for these issues in this research?
2. The authors only considered degree days (DD) as a proxy for energy consumption. However, what about dewpoint temperature or accounting for humidity in the energy consumption? Research has shown that dewpoint temperature is a better predictor of climate induced energy demand (Alipour et al. 2019; Mukherjee et al. 2019; Mukherjee and Nateghi 2017, 2018; Nateghi and Mukherjee 2017; Raymond et al. 2019). This is because humidity contributes to the feels like temperature, which cannot be solely explained by the surface temperature. Moreover, since this paper presents a global study, humidity and dew point temperature will vary significantly in the polar, temperate and tropical regions; this, in turn will have a significant variation in energy demand that cannot be only captured by the heating or cooling degree days. I would like to see a detailed explanation on how the authors have accounted for this humidity induced variability in the energy demand?
3. The authors rightly pointed out that the calculation of the degree days (cooling and heating) is based on a baseline temperature that is different for different countries. To test whether the change in base temperature affects the trends in HDD or CDD, the authors also used a different base temperature. However, I am still not convinced by this method. The reason being, the climatic conditions of U.S. and U.K are not that different when compared to an arid climate in Africa or a humid tropical climate like India. For example, the cooling temperature threshold which is considered as 18.3 °C in U.S. or 22°C in U.K. are lower than the average winter temperatures in some of the cities such as Mumbai, India with average winter temperature as 27.2°C. However, occupants of the building do not use air conditioning because it's comfortable temperature for them. Thus, using a single baseline temperature for all the cities across the world cannot capture the true trend in cooling or heating energy consumption. It would be more appropriate if different baseline temperatures, specific to the countries / cities are used for the DD calculation purpose.
4. In Supplement (pg 16, lines 259-260), why the correlation between the U.S. and U.K. methods is not shown? This is an important aspect of the research and I would recommend to include that in the manuscript.
5. In the supplement, (Pg. 2 line 59-61) the authors mention – "The assumption of the DD method is that it is the daily mean temperature, sometimes in combination with daily minimum and maximum temperatures, is a good proxy of the cumulated temperature during one day, and thus of the energy demand."—This statement is dubious and the such an assumption is a very strong one. There are several modeling techniques that are being used to model the energy demand and DD nexus. First, this is only the climate-sensitive portion of the demand. It doesn't account for any non-climate induced energy demand such as energy needed for agriculture, transportation, etc.

i.e., this energy that the authors are referring to is only that portion of energy that is needed for space conditioning (i.e., heating or cooling).
6. Although the paper title states "Large uncertainties in heating and cooling energy demand under climate change", the authors do not show any modeling approach to estimate how the energy change is affected by the climate change. This is one of my major concerns. Climate change does not only refer to increase in temperature but refers to the shift in climate in actual sense. Thus, besides heating and cooling degree days (which is a derived climate indicator for temperature), energy demand in also affected by shifts in precipitation or windspeed. Sometimes, due to this shift, the increase in energy demand owing to higher surface temperatures can be offset by the cooling effect of higher precipitation or windspeed, as the latter two climate variables are inversely related to an increasing energy demand (Mukherjee and Nateghi 2017). Hence, I am not convinced that the title is appropriate for this study. In my perspective, proportional increase / decrease in heating / cooling degree days cannot appropriately reflect the proportional increase or decrease in energy demand.
7. In Section II of the supplement, (Pg 6., lines 104-109), the authors classified the entire global climate into three main climatic zones. What is the basis for this classification? Please include relevant reference for this classification.
8. In summary, I would suggest, instead of saying this study is an analysis of energy demands, I would say restructure the manuscript in a way that it focuses on degree days analysis. Besides temperature, several other uncertainties are associated with future energy demand under climate change and just saying the it is proportional to the heating and cooling degree days doesn't do proper justice. Such simplistic assumption might render the results to be highly biased.

References:
Alipour, P., Mukherjee, S., and Nateghi, R. (2019). "Assessing climate sensitivity of peak electricity load for resilient power systems planning and operation: A study applied to the Texas region." Energy.
Mukherjee, S., CR, V., and Nateghi, R. (2019). "Evaluating regional climate-electricity demand nexus: A composite Bayesian predictive framework." Applied Energy, (under review).
Mukherjee, S., and Nateghi, R. (2017). "Climate sensitivity of end-use electricity consumption in the built environment: An application to the state of Florida, United States." Energy, Elsevier, 128, 688–700.
Mukherjee, S., and Nateghi, R. (2018). "A data-driven approach to assessing supply inadequacy risks due to climate-induced shifts in electricity demand." Risk Analysis, under 2nd review.
Nateghi, R., and Mukherjee, S. (2017). "A Multi-Paradigm Framework to Assess the Impacts of Climate Change on End-Use Energy Demand." PloS one, 12(11), e0188033.
Raymond, L., Gotham, D., McClain, W., Mukherjee, S., Nateghi, R., Preckel, P. V, Schubert, P., Singh, S., and Wachs, E. (2019). "Projected climate change impacts on Indiana's Energy demand and supply." Climatic Change.

Reviewer #2 (Remarks to the Author):

The manuscript "Large uncertainties in heating and cooling energy demand under climate change" calculates heating- and cooling-degree days on a global grid using temperature projections from a standard set of climate models, then analyses the variation between models and compares their past and future trends. Although the calculation itself is trivial, the study is well conducted, the analysis is insightful, and the results are both well presented and credible. In my opinion, this research is of high quality.

However, I do have some specific concerns, and also some unsolicited suggestions for the authors.

Although I find the study very interesting, I am a little concerned that the study might appeal to a somewhat narrow audience. However, I admit that I am not an expert at such matters, and defer this question to the editors, who will have a much better understanding of this question.

Another concern I have is about the choice to focus almost entirely on relative changes in the

results from the section "Trends in energy demand" and onwards. I am not convinced it is such a sensible choice. Firstly, it is not clear to me how the relative changes is calculated when the denominator is zero. Secondly, the scale of the changes can get confusing, as a change from 1 CDD to 2 CDD will
look more significant than a change from 1000 CDD to 1900 CDD. Since discussing relative changes and absolute changes have each their advantages and disadvantages, it would perhaps be a good idea to use both, and not exclusively choose one over the other.

Reading the manuscript, I was somewhat disappointed: the summary promises a "novel indicator", but I did not see any novel indicators. I am not sure what the authors are referring to, since neither HDD/CDD nor multi-model means are particularly novel. In the end, I think perhaps it was a poor choice of words in the summary that oversold it a little? If not, could the authors perhaps tell me
which of the indicators is supposedly novel?

Finally, I suggest revising the manuscript with a stronger focus on clarity. Many sentences are very involved -- especially some of the "elevator analysis" sentences that present increases and decreases -- and I had to read them several times before understanding what quantities were actually increasing and decreasing. I think potential future readers would greatly appreciate it if the authors could revise the manuscript with this in mind.

Ian Michael Trotter

Reviewer comments

Reviewer #1 (Remarks to the Author):

The paper presents an analysis of how the uncertainties of climate models affect the energy demand projections globally in a warming world. Authors analyzes various scenarios and reports the results on heating and cooling proxies, global patterns in heating and cooling, trends in energy demands, inter-model variability, by the end of the century energy demands and robustness of the trends. The paper is well written, but I have major concerns regarding some of the basic concepts.

We thank Reviewer #1 for raising the issues below, which mainly concern the use of Degree-Days to estimate the future energy demand. We recognize that our focus has not been well explained in the previous version. Indeed we aim at analyzing only the climate-driven energy demand trends and the uncertainties in their projection.

Even if other proxies of the climate-driven energy demand could be used, we assume that the Degree-Days methodology is relevant for our goal because it is (i) physically comprehensive proxy (temperature-based), (ii) a historical method, (iii) still used nowadays by energy companies, and (iv) largely published, as shown by these selected examples:

- *Yalew et al., 2020 (Nature Energy; 5 citations on 2/2/2021)*
- *Van Ruijven et al., 2019 (Nature Communications; 54 citations on 2/2/2021)*
- *Hasegawa et al., 2016 (Palgrave Communications, 20 citations on 2/2/2021)*
- *Wang and Chen, 2014 (Energy and Buildings; 193 citations on 2/2/2021)*
- *Issac and Van Vuuren, 2009 (Energy Policy, 830 citations on 2/2/2021)*
- *Amato et al., 2005 (Climate Change, 264 citations on 2/2/2021)*

First, we do not intend to neglect the complexity of predicting end-use energy demand, and we agree with Reviewer #1 on the important role of non-climatic factors (socio-economic and technological), as well as of other climate variables besides temperature. It was already mentioned in the first submission (L47-49, L130-133, L238-240) but a stronger emphasis was needed from the introduction onward. This is now done in the revised manuscript.

Second, it is not our goal to provide an alternative approach to statistical models which integrate socio-economic, technological and climatic components to predict end-use energy demand. Rather, our investigation focuses on the *climate-driven* component of energy demand. The reason for this focus on climate is that other studies about future energy demand have neglected the uncertainties in temperature projections from climate model simulations. Our aim is to put forward these

uncertainties focusing on climate-driven energy demand trends, expressed as relative changes in heating and cooling degree days.

The justifications for our focus have been clearly explained in the new version. Numerous modifications and additions to the manuscript were made accordingly (notably in the *Title, Introduction, Methods* and *Supplementary Information*) to address the specific concerns of Reviewer #1 outlined below. Finally, our changes have no impact on the conclusion that a precise quantification of future energy demand for heating and cooling buildings must take into account the large uncertainties in temperature projections.

1. In page 2 of the Supplement, (Pg. 2, line 38-39), the authors conclude that the relative change in the heat flux (F) between the inside and the outside of a building is equivalent to the relative change in temperature. However, this change in heat flux is independent of the building's properties only when the building characteristics don't change over time. This assumption is a strong one, especially when long-term energy demand forecast is referred to. For example, the efficiency of buildings is changing fast along the increase in efficiency of the equipment such as air conditioner or heaters. Moreover, there is also a shift in the type of energy (for example, electricity, natural gas, renewables) being used for space cooling or heating. Thus, this is a very important context and I want to see how the authors are accounting for these issues in this research?

Response:

We thank Reviewer #1 for pointing out this shortcoming. The focus of our work is only on the climate-driven trends in energy demand for the heating and cooling of buildings. It is not meant as a prediction of the end-use energy demand, which would require a prediction of many other factors. These climate-driven trends, i.e. the changes over time related to climate change, can be interpreted regardless of the actual end-use energy demand in a given place.

While we were aware of the relevance of non-climatic factors (cf. first submission L47-49, L130-133, L238-240), our focus on climate had not been stated clearly enough. Indeed, we do not expect that building properties, the efficiency of heating and cooling systems, nor the type of energy will remain constant in the future. On the contrary, we agree with Reviewer #1 that these factors are likely to change over the time scale considered. Additionally, future population growth, economic growth, policy change and technological advances will have a significant impact on end-use energy demand.

The basis for our study is the analysis of climate using temperature-based proxies of the energy demand. Our research highlights the changes in these proxies under

climate change, and identifies the uncertainties related to the variability in CMIP5 climate projections. The consequences of our findings provide insights for climate change adaptation, e.g., through changes in building properties, energy systems efficiency and energy production.

Changes in the manuscript:

A number of changes were made to better emphasize our focus on climate-driven changes in energy demand and to acknowledge the relevance of non-climatic factors:

a) In the *Introduction*, the non-climatic factors are specified in more detail (L42-48) as well as the influence of different climatic factors on the energy demand and the use of the Degree Days method in this context (L58-64).

The energy demand for heating and cooling buildings is driven by a climatic component, a socio-economic component (population density and behavior of people, gross domestic product, price of energy) and by a technological component (design and material determining the thermal properties of the building, efficiency of heating and cooling systems) (Isaac and van Vuuren, 2009; Trotter et al., 2016; Urge-Vorsatz et al., 2013). In addition to long-term trends in these three components, there is a short-term variability in energy demand, and in related CO_2 emissions, which is mostly linked to climate variability (Bréon et al., 2017; Isaac and van Vuuren, 2009).

The Degree-Days method (hereafter DD) is the historical method for estimating the heating and cooling energy demand (Day, 2006; Thom, 1959, 1954) (*cf.* Methods section). A key assumption of this method is that the average temperature of a day provides a good proxy for the human thermal discomfort (Thom, 1959, 1954), and thus of the daily energy demand (Day, 2006; Valor et al., 2001; Wang and Chen, 2014). The DD represent the difference between the outside daily temperature and the range of comfortable indoor temperatures. In other words, the DD is the cumulated temperature during one day below a base temperature, the so-called Heating Degree-Days (HDD); and above a base temperature, the so-called Cooling Degree-Days (CDD).

b) In the *Discussion*, we highlight that with temperature alone, we cannot estimate end-use energy (L240-243).

Other climatological factors, such as precipitation or wind, are important as well to estimate the future end-use energy demand (Nateghi and Mukherjee, 2017). However these factors are physically linked to the temperature change, therefore focusing on the projected temperature, our study constitutes a necessary first step.

c) In the *Methods*, we further explain the limitations (L277-280) and the relevance (L365-369) of our approach. .

Although the integration of other climatic (cloud, wind, precipitation, snow) and non-climatic (socio-economical or technological) variables are needed to accurately estimate the end-use energy demand

of buildings (Mukherjee et al., 2019; Nateghi and Mukherjee, 2017), the temperature is the main climate driver of energy demand for building heating and cooling (Apadula et al., 2012).

Conceptually, our methodology allows comparing the potential climate-driven energy demand trends for heating or for cooling of an exact same building (structure, materials, heating and cooling system) that would be located anywhere in the world. For a specific location, the trends in HDD and in CDD represent how the energy demand of this hypothetical building is projected to change in time due to the climate.

d) In the *Supplementary Information*, we removed the sub-section "*Thermodynamical basics*" dealing with a schematic view of our concept. Although it aimed at illustrating the DD concept, it was misleading because the presented equations were not meant to provide a calculation to model a building's energy consumption.

2. The authors only considered degree days (DD) as a proxy for energy consumption. However, what about dewpoint temperature or accounting for humidity in the energy consumption? Research has shown that dewpoint temperature is a better predictor of climate induced energy demand (Alipour et al. 2019; Mukherjee et al. 2019; Mukherjee and Nateghi 2017, 2018; Nateghi and Mukherjee 2017; Raymond et al. 2019). This is because humidity contributes to the feels like temperature, which cannot be solely explained by the surface temperature. Moreover, since this paper presents a global study, humidity and dew point temperature will vary significantly in the polar, temperate and tropical regions; this, in turn will have a significant variation in energy demand that cannot be only captured by the heating or cooling degree days. I would like to see a detailed explanation on how the authors have accounted for this humidity induced variability in the energy demand?

Response:

We agree with reviewer #1 that humidity is a relevant variable to study the climate-driven energy demand. Temperature is the main climatic driver of the energy demand and more precisely its combination with humidity (such as dew point). We include some dependence on humidity by applying the UK Met Office calculation of DD, which uses not only the daily mean, but also daily minimum and maximum temperatures.

There is a clear relationship between dew point temperature and the diurnal temperature range. For a given daily mean temperature, the daily minimum and maximum are close ($T_{min} \approx T_{max}$) in wet environments and the dew point is close to the ambient temperature, while in dry environments, the amplitude of the diurnal temperature cycle is large ($T_{min} << T_{max}$) and the dew point is well below the ambient temperature.

The reference given by Reviewer #1 comparing dew point temperature to DD calculated with the US method (based on daily mean temperature and not taking into account the diurnal temperature range), conclude that dew point temperature is a better predictor for energy demand. However, the suggested study of *Nateghi and Mukherjee 2017 (PLOSone)* uses Tmin and Tmax as predictors of future end-use energy demand, because dew point temperature is not available from the climate projection. The dew point temperature could in principle be calculated from temperature and humidity, but humidity is not available at a daily time step from most of the 30 climate model simulations used in our study.

Changes in the manuscript:

We underline the relevance of humidity besides temperature for energy demand, and we explain in more detail it influence on the UK calculation of DD that we apply.

a) In the *Introduction*, we explain the relation between humidity and the diurnal temperature range (L49-57).

Among the climate variables that influence the energy demand, ambient temperature is prominent (Apadula et al., 2012), or more precisely its combination with humidity (Mukherjee et al., 2019). The minimum and maximum daily temperatures are good predictors of the energy demand (Nateghi and Mukherjee, 2017) as they represent the diurnal cycle of ambient temperature. The amplitude of this diurnal cycle is large in dry areas and small in wet areas. The day-to-day variability in energy demand depends on temperature following a V-shape curve with a minimum related to human thermal comfort as well as other socio-economic and technological factors (De Cian and Sue Wing, 2019; Valor et al., 2001). This minimum is found for a similar daily mean temperature around 16 °C, even over a large area like Europe (Wenz et al., 2017). Therefore, a comprehensive analysis relating the trends in the projected temperature and its consequences on energy demand is possible.

b) In the *Discussion*, we acknowledge the need for further research in this direction (L235-240).

We made use of a metric derived from cumulated surface temperature to estimate the trends of climate-driven energy demand. The surface temperature is the most important determining factor for this demand (Apadula et al., 2012). Atmospheric humidity is also a key factor (Maia-Silva et al., 2020). Our methodology includes some dependence on humidity, through the use of the daily minimum and maximum temperatures. Nevertheless, further research is needed for an explicit inclusion of humidity in the estimation of the future energy demand.

c) In the *Methods* section, we provide references for the role of human discomfort in the development of DD applications in building heating and cooling (L264-277).

Proxies of climate-driven energy demand for heating and cooling buildings

Surface temperatures used to be monitored at a daily time step before the digitization of meteorological data. Consequently, methods to estimate energy demand have also been based on temperature data with a daily time step. The Degree-Days (*hereafter* DD) method has long been used

to estimate heating and cooling energy demand (Day, 2006), and relies on the link between human discomfort sensation and temperature variability (Thom, 1959, 1954). The main assumption of the DD method is that the annual cumulative temperature above a temperature threshold (called base temperature, *Tbase*) of the daily mean temperature only (that we refer to as US calculation, Table 2), or in combination with daily minimum and maximum temperatures (that we refer to as UK calculation, Table 1), is a good proxy of the climate-driven energy demand for heating and cooling buildings (Day, 2006; Wang and Chen, 2014). The UK calculation based on four day types (*cold day, mostly cold day*, *mostly warm day* or *warm day)* is more adequate to analyze energy demand in different climate regions (Day, 2006; Thom, 1954). This is because it takes into account differences in the amplitude of the diurnal temperature cycle.

3. The authors rightly pointed out that the calculation of the degree days (cooling and heating) is based on a baseline temperature that is different for different countries. To test whether the change in base temperature affects the trends in HDD or CDD, the authors also used a different base temperature. However, I am still not convinced by this method. The reason being, the climatic conditions of U.S. and U.K are not that different when compared to an arid climate in Africa or a humid tropical climate like India. For example, the cooling temperature threshold which is considered as 18.3 °C in U.S. or 22°C in U.K. are lower than the average winter temperatures in some of the cities such as Mumbai, India with average winter temperature as 27.2°C. However, occupants of the building do not use air conditioning because it's comfortable temperature for them. Thus, using a single baseline temperature for all the cities across the world cannot capture the true trend in cooling or heating energy consumption. It would be more appropriate if different baseline temperatures, specific to the countries / cities are used for the DD calculation purpose.

Response:

We agree with Reviewer #1 that the choice of base temperature has an influence on the calculated values of HDD and CDD, and that the comfortable temperature range depends on people's habits (Steemers and Yun, 2009). We also agree that a single base temperature "*cannot capture the true trend in energy consumption*", because the prediction of the actual consumption will depend on the choice of this base temperature. Our goal is to investigate climate-driven trends in energy demand for heating and cooling buildings (*cf.* Response to comment 1). We show in a new section of the *Supplementary Information* that these trends are similar regardless of the choice of the base temperature.

The DD method is used to estimate building energy demand in tropical and subtropical climates like India (Borah et al., 2015). The continental-scale HDD and CDD investigations by Spinoni et al. (2018) and Petri & Caldeira (2015) also use spatially uniform base temperatures. Applying country-specific base temperatures in a global study is not adequate to study the uncertainties related to climate change, because it would lead to breaks in the HDD and CDD patterns along country borders

instead of displaying climatic gradients. Furthermore, data to adjust country-specific energy demand predictions by the behavior of the local population are not available.

Changes in the manuscript:

In order to demonstrate the low influence of the choice of the base temperature on the trends in the heating and cooling proxies, a comparison of different base temperatures has been added in the *Supplementary Information (*Section IV. Sensitivity of HDD and CDD trends to the choice of base temperature […]*)*. Correlation coefficients are now provided for HDD and CDD calculated with both base temperatures (for the three studied time periods). We have added a new figure with difference maps for the heating and cooling proxies calculated with different base temperatures (Figure S15, see below). These additions illustrate that the differences are constant in time (for the multi-model mean), and consequently the trends in the heating and cooling proxies are similar when altering the base temperature.

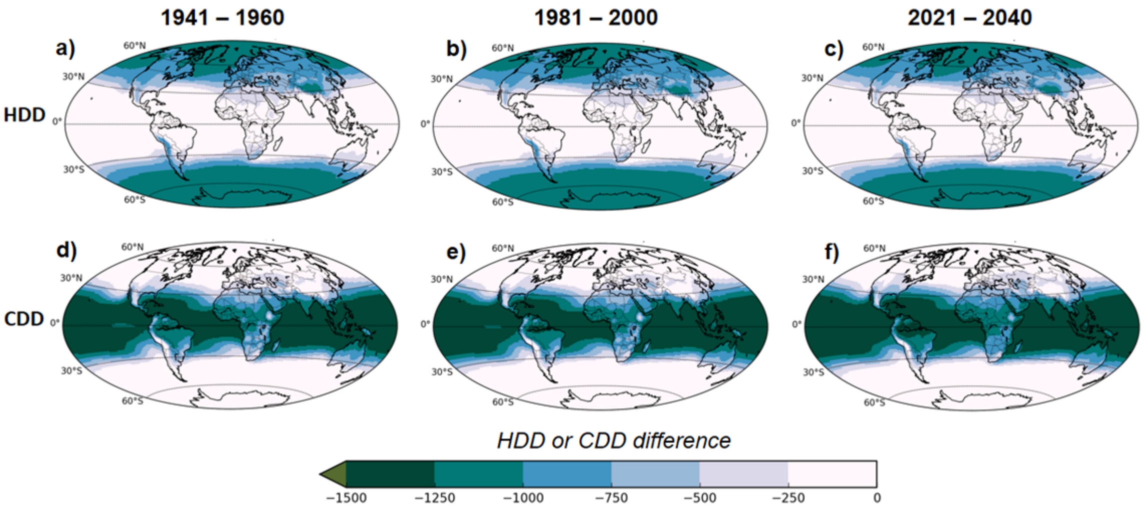

Figure S15: Differences of HDD calculated with a base temperature (Tbase) of 15.5 °C and HDD calculated with Tbase of 18.3 °C and differences of CDD calculated with Tbase of 18.3 °C and CDD calculated with Tbase of 22 °C. *The differences are shown for 20-year averages of (**a**, **b**, **c**) annual HDD, and (**d**, **e**, **f**) CDD, for the periods 1941-1960, 1981-2000, and 2021-2040.*

4. In Supplement (pg 16, lines 259-260), why the correlation between the U.S. and U.K. methods is not shown? This is an important aspect of the research and I would recommend to include that in the manuscript.

Response:

This aspect is indeed important. In the first submission we showed that our conclusions are the same with UK and US calculation, from the comparison of the spatial patterns of the trends in the heating and cooling proxies and the inter-model variability (Figure S8). In the revised manuscript, we extend this analysis and we add a new figure in the *Supplementary Information*.

Changes in the manuscript:

A more comprehensive comparison of the US and UK calculation methods is included to the *Supplementary Information* (Section IIIa […]). We give the correlation coefficients for HDD (resp. CDD) calculated with the different methods (0.99 for HDD and 0.97 for CDD), which are identical for the three studied time periods. We further add a new figure (Figure S7a-f, see below) showing the differences between the UK and US methods with maps of HDD and CDD. We conclude that the difference between the methods is constant in time, thus the projected trends and the inter-model variability in the heating and cooling proxies are not sensitive to the calculation method.

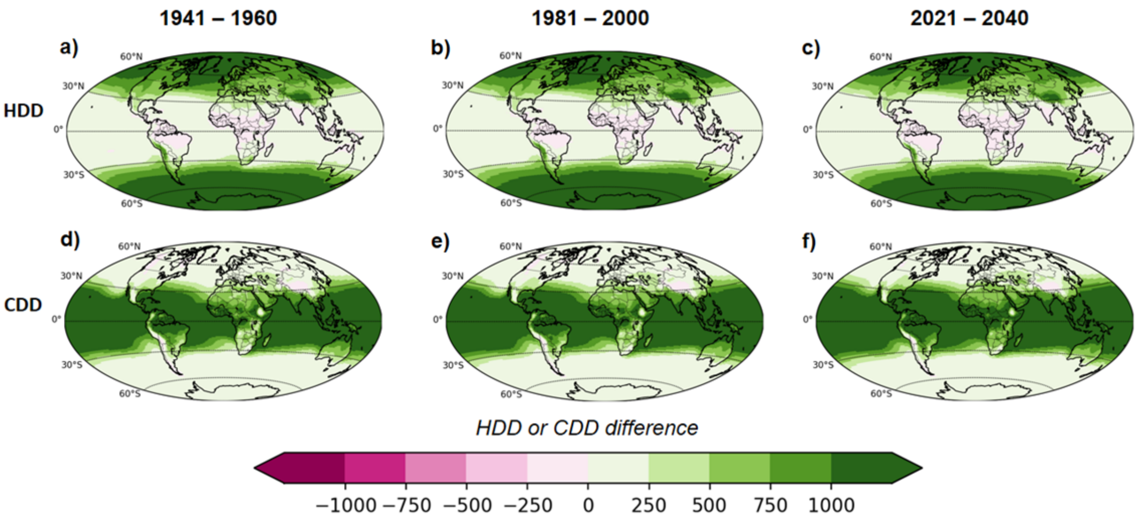

Figure S1: Differences of Heating Degree Days (HDD) resp. Cooling Degree Days (CDD) calculated with the UK Met Office method and HDD resp. CDD calculated with the US standard method. *The differences are shown for 20-year averages of (**a**, **b**, **c**) annual HDD, and (**d**, **e**, **f**) annual CDD, for the periods 1941-1960, 1981-2000, and 2021-2040.*

5. In the supplement, (Pg. 2 line 59-61) the authors mention – "The assumption of the DD method is that it is the daily mean temperature, sometimes in combination with daily minimum and maximum temperatures, is a good proxy of the cumulated

temperature during one day, and thus of the energy demand."—This statement is dubious and such an assumption is a very strong one. There are several modeling techniques that are being used to model the energy demand and DD nexus. First, this is only the climate-sensitive portion of the demand. It doesn't account for any non-climate induced energy demand such as energy needed for agriculture, transportation, etc. i.e., this energy that the authors are referring to is only that portion of energy that is needed for space conditioning (i.e., heating or cooling).

Response:

We are especially grateful to Reviewer #1 for pointing out this shortcoming of our previous manuscript about the denomination of the quantity that we analyze. It is true that our study investigates the trends in the climate-driven energy demand for heating and cooling buildings using proxies. We have changed the entire manuscript using this denomination to avoid any confusion.

Changes in the manuscript:

In the new submission, the statement pointed out by Reviewer #1 appears in the *Methods* section, modified as follows (L268-274):

The Degree-Days (*hereafter* DD) method has long been used to estimate heating and cooling energy demand (Day, 2006), and relies on the link between human discomfort sensation and temperature variability (Thom, 1959, 1954). The main assumption of the DD method is that the annual cumulative temperature above a temperature threshold (called base temperature, *Tbase*) of the daily mean temperature only (that we refer to as US calculation, Table 2), or in combination with daily minimum and maximum temperatures (that we refer to as UK calculation, Table 1), is a good proxy of the climate-driven energy demand for heating and cooling buildings (Day, 2006; Wang and Chen, 2014).

6. Although the paper title states "Large uncertainties in heating and cooling energy demand under climate change", the authors do not show any modeling approach to estimate how the energy change is affected by the climate change. This is one of my major concerns. Climate change does not only refer to increase in temperature but refers to the shift in climate in actual sense. Thus, besides heating and cooling degree days (which is a derived climate indicator for temperature), energy demand in also affected by shifts in precipitation or windspeed. Sometimes, due to this shift, the increase in energy demand owing to higher surface temperatures can be offset by the cooling effect of higher precipitation or windspeed, as the latter two climate variables are inversely related to an increasing energy demand (Mukherjee and Nateghi 2017). Hence, I am not convinced that the title is appropriate for this study. In my perspective, proportional increase / decrease in heating / cooling degree days cannot appropriately reflect the proportional increase or decrease in energy demand.

Response:

The major concern expressed by Reviewer #1 is related to the imprecise previous denomination of the proxies that we use. It is now clearly stated throughout the manuscript that this study analyzes the climate-driven trends in energy demand using Heating and Cooling Degree Days. This study does not analyze the end-use energy demand, rather it focuses on climate-driven energy demand trends for heating and cooling buildings.

We think that the use of temperature-based proxies is a necessary first step to study, using climate model projections, the future evolution of the climate-driven energy demand - with uncertainties - at the global scale. Consequently, we changed the title to better reflect our focus on the climate-driven trends in the energy demand.

Changes in the manuscript:

We changed the title to:

"Large uncertainties in trends of energy demand for heating and cooling under climate change"

Furthermore, several modifications were made throughout the manuscript to re-word sentences which could have suggested a too simplistic interpretation of the link between energy demand and climate (see replies to comments 1, 2 and 5).

7. In Section II of the supplement, (Pg 6., lines 104-109), the authors classified the entire global climate into three main climatic zones. What is the basis for this classification? Please include relevant reference for this classification.

Response:

While tropical, temperate and polar regions are commonly used expressions, we agree that they are not clearly defined in the previous version of the manuscript. In order to use a more obvious classification for a global overview of the heating and cooling proxies ranges, we define three zones based on latitude:

(i) High-latitude (above 60°N or below 60°S)
(ii) Mid-latitude (from 60°N to 30°N; or from 60°S to 30°S)
(iii) Inter-tropical (from 30°N to 30°S)

Using this classification, the comment of Figure S1 has been improved. We thank Reviewer #1 for this remark.

Changes in the manuscript:

In the main article, the sentence referring to these climatic zones (L105-110) is changed accordingly:

Our results show typical values of the heating proxy over land areas between 0 and 1500 HDD in inter-tropical regions (from 30°N to 30°S), between 1500 and 5000 HDD in mid-latitude regions (from 60°N to 30°N; or from 60°S to 30°S) and above 5000 HDD in polar regions (above 60°N or 60°S). Values of the cooling proxy are between 400 and 2000 CDD in inter-tropical regions, and between 0 and 400 CDD in mid-latitudes. These values change in a warming world. However the magnitude of the changes is not globally uniform (Fig. S1).

In the *Supplementary Information*, the text referred to by Reviewer #1 is now in Section I. The climatic zones are specified by adding the latitude ranges.

The global HDD patterns (Fig. S**Error! Reference source not found.** a, b, c) correspond to three main climatic zones, the polar regions above 60°N and 60°S (HDD > 5000), the mid-latitude regions from 60°N to 30°N and from 60°S to 30°S (1000 < HDD < 5000) and the tropical regions between 30°N and 30°S (HDD < 1000).

8. In summary, I would suggest, instead of saying this study is an analysis of energy demands, I would say restructure the manuscript in a way that it focuses on degree days analysis. Besides temperature, several other uncertainties are associated with future energy demand under climate change and just saying it is proportional to the heating and cooling degree days doesn't do proper justice. Such simplistic assumption might render the results to be highly biased.

Response:

We recognize that many other factors - with their uncertainties - must be taken into account in order to quantify future end-use energy demand. Developing adequate climate change mitigation strategies in the building sector, e.g. through adaptation of building properties or the efficiency of climatization systems, requires knowledge of the temperature-dependent trends, and their uncertainties. Our study focuses on the trends in the climate-driven energy demand, which is now well explained through a number of modifications in the entire manuscript.

Overall, the changes that we made based on the valuable comments of Reviewer #1 have improved the clarity of the manuscript, and reinforced our conclusions: Future climate change will reduce the climate-driven energy demand for heating and increase the climate-driven energy demand for cooling globally. While the direction of the changes is clear, their magnitude remains highly uncertain due to the variability in temperature projections among individual simulations.

References:

Alipour, P., Mukherjee, S., and Nateghi, R. (2019). “Assessing climate sensitivity of peak electricity load for resilient power systems planning and operation: A study applied to the Texas region.” Energy.

Mukherjee, S., CR, Vineeth, and Nateghi, R. (2019). “Evaluating regional climate-electricity demand nexus: A composite Bayesian predictive framework.” Applied Energy, (under review).

Mukherjee, S., and Nateghi, R. (2017). “Climate sensitivity of end-use electricity consumption in the built environment: An application to the state of Florida, United States.” Energy, Elsevier, 128, 688–700.

Mukherjee, S., and Nateghi, R. (2018). “A data-driven approach to assessing supply inadequacy risks due to climate-induced shifts in electricity demand.” Risk Analysis, under 2nd review.

Nateghi, R., and Mukherjee, S. (2017). “A Multi-Paradigm Framework to Assess the Impacts of Climate Change on End-Use Energy Demand.” PloS one, 12(11), e0188033.

Raymond, L., Gotham, D., McClain, W., Mukherjee, S., Nateghi, R., Preckel, P. V, Schubert, P., Singh, S., and Wachs, E. (2019). “Projected climate change impacts on Indiana’s Energy demand and supply.” Climatic Change.

Reviewer #2 (Remarks to the Author):

The manuscript "Large uncertainties in heating and cooling energy demand under climate change" calculates heating- and cooling-degree days on a global grid using temperature projections from a standard set of climate models, then analyses the variation between models and compares their past and future trends. Although the calculation itself is trivial, the study is well conducted, the analysis is insightful, and the results are both well presented and credible. In my opinion, this research is of high quality.

However, I do have some specific concerns, and also some unsolicited suggestions for the authors.

1 - Although I find the study very interesting, I am a little concerned that the study might appeal to a somewhat narrow audience. However, I admit that I am not an expert at such matters, and defer this question to the editors, who will have a much better understanding of this question.

Response:

We thank Reviewer #2 for his interest in our study. The major interest of our methodology is to integrate the Human dimension in climate change analysis, because we look at cumulative temperature above or below a threshold based on human comfort perception, which has consequences for the energy demand for heating and cooling buildings.

The study of climate-driven energy demand trends for heating and cooling buildings has important socio-economic implications related to the future of end-use energy demand. It has implications for architecture and building design, city planning, economics, energy production and related greenhouse gas emissions, and policy development. Our study is therefore relevant for a large interdisciplinary audience.

We show that it is necessary to take into account the uncertainties in heating and cooling trends linked to temperature projections. For example in Sao Paulo (Brazil), the trend in our cooling proxy is projected to increase between 10% and 100% from 1981-2000 to 2021-2040, depending on individual climate model simulations.

Changes in the manuscript:

To underline the importance of the study, we modified the *Discussion* as followed (L244-255):

Reliable information is needed by individuals, city planners, policy makers and companies to manage or mitigate future changes in energy demand. The trends in the heating and cooling needs of buildings are particularly useful to indicate which regions will likely experience large changes, and thus benefit most from improvements in thermal insulation and the efficiency of heating/cooling systems. For example, we show that the increasing trend in cooling for Paris (most analyzed mid-latitude cities present comparable results) is projected to reach +80% in the multi-model mean, leading to a potential massive adoption of cooling systems. However, this number alone is not sufficient to assess whether such measures will indeed be needed, because the increase may be as small as +2% or as large as +348% according to individual model estimates. The quantification of the increasing trend in cooling is thus highly uncertain and highlights the need to take the inter-model variability into account when designing adaptation plans, whether they concern architecture, the efficiency of climatization systems, or power generation and networks.

We further added to the *Methods* a statement about the applicability of the concept (L388-391):

Conceptually, our methodology allows comparing the potential climate-driven energy demand trends for heating or for cooling of an exact same building (structure, materials, heating and cooling system) that would be located anywhere in the world. For a specific location, the trends in HDD and in CDD represent how the energy demand of this hypothetical building is projected to change in time due to the climate.

2 - Another concern I have is about the choice to focus almost entirely on relative changes in the results from the section "Trends in energy demand" and onwards. I am not convinced it is such a sensible choice. Firstly, it is not clear to me how the relative changes is calculated when the denominator is zero. Secondly, the scale of the changes can get confusing, as a change from 1 CDD to 2 CDD will look more significant than a change from 1000 CDD to 1900 CDD. Since discussing relative changes and absolute changes have each their advantages and disadvantages, it would perhaps be a good idea to use both, and not exclusively choose one over the other.

Response (to the first point):

We thank Reviewer #2 for pointing out the missing explanation. Obviously, a relative difference cannot be calculated when HDD or CDD of the reference period, i.e. the denominator, is zero. Therefore, we assign *Not a Number* (NaN) to the grid cells with a denominator <1. The same is done when the absolute differences in HDD or CDD between two time periods are insignificant according to our statistical test. These grid cells with NaN values are displayed by the gray shading in Fig. 2.

In the case of HDD, this does not have any influence on the result. When the value of the (earlier) reference period is zero, the value of the later period, and

consequently the difference of the two periods is also zero. This is because the 20-year HDD averages decrease everywhere on the globe between our studied time periods, and grid cells with 0 HDD in the past, therefore have 0 HDD in the present and future.

In the case of CDD, this leads to grid cells with NaN at the margins of the areas with insignificant changes. Excluding denominators <1 removes grid cells from the analysis where cooling demand is very low despite a large relative increase between two periods. For example, if CDD in the past was 0.01, and the difference with the following period was 3, the relative change would be high, although the absolute change of <3 CDD is negligible.

Changes in the manuscript:

The explanation of how denominators <1 are dealt with in the calculation of relative HDD and CDD changes has been added to the *Methods* section (L351-364).

Calculation of trends

The absolute differences in HDD and CDD values are calculated between the 1981-2000 and 1941-1960 averages, referred to as "*past*" changes, and between the 2021-2040 and 1981-2000 averages, referred to as "*future*" changes, for each model and for the MMM (Fig. 1). Heating and cooling trends are expressed in terms of relative HDD and CDD differences, in percentage compared to the earlier period, shown for the MMM (Fig. 2). A Student t-test (using two dependent data samples) is applied to test whether the averages of the thirty simulations are significantly different over two compared time periods at a 95% confidence interval.

In order to compute the relative differences in HDD and CDD values, *i.e.* our proxy of the climate-driven energy demand trends, two conditions are used:

- The absolute differences in HDD and CDD values must be significant according the statistical test;
- The annual average in HDD and CDD values for the earlier period which is the denominator of the relative differences, must be greater than 1.

Response (to the second point):

We agree with Reviewer #2 that relative differences (i.e. trends) must be analyzed together with absolute differences (as already stated in the previous version of the manuscript L152-156). We put a stronger focus on relative HDD or CDD change because we want to study the climate-driven trends in our proxies of the energy demand represent. Of course, the meaning of e.g. a 10% change in the cooling proxy is different depending on the absolute values, but it can be interpreted as the local climate-driven trend in cooling. The example the reviewer gave illustrates that it

is important to show both absolute and relative changes side by side to better evaluate the meaning of a certain change expressed in percentage.

Changes in the manuscript:

The new Figure 1 (see below) showing absolute changes in heating and cooling has been added to the manuscript.

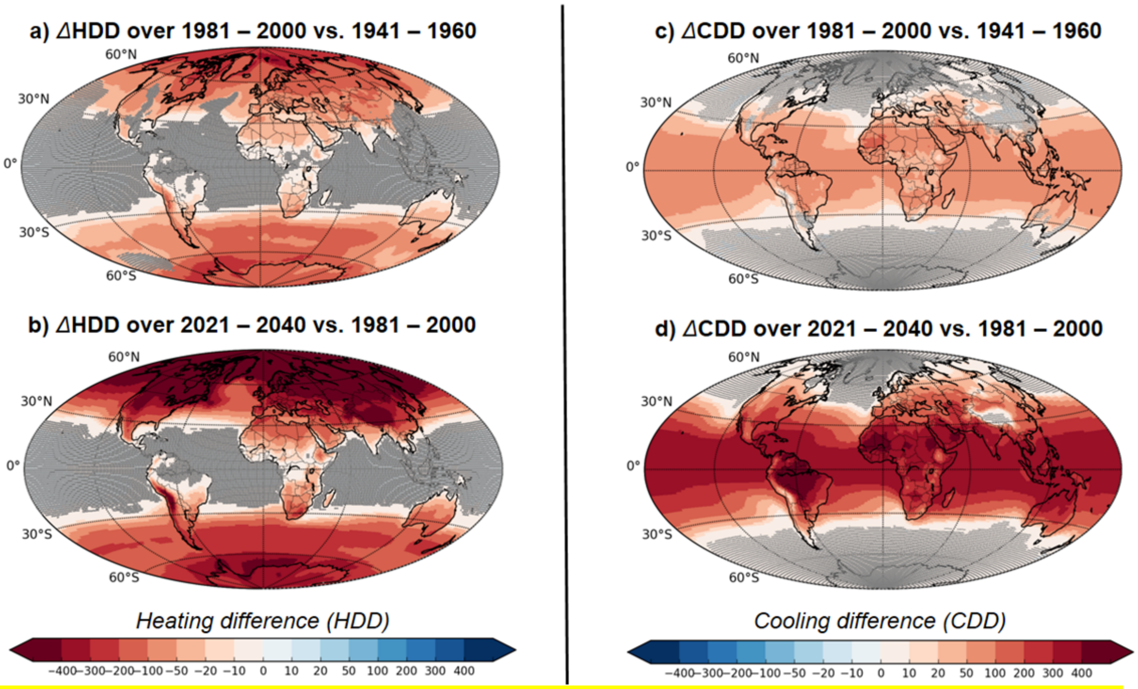

***Figure 1: Global climate-driven changes in energy demand for heating and cooling buildings.** The quantification of the change is expressed as absolute differences in annual (left) Heating Degree Days (ΔHDD) and (right) Cooling Degree Days (ΔCDD) between the periods (panels **a, c**) 1981-2000 and 1941-1960, and (panels **b, d**) 2021-2040 and 1981-2000. Multi-model means of annual HDD and CDD sums were calculated using the daily mean, minimum and maximum temperatures from 30 CMIP5 models, and averaged over the 20-year periods. The areas shaded in gray indicate locations where the difference is not significantly different from zero according to Student t-test at a 95% confidence interval. Projections are based on the RCP8.5 scenario. Note that the color scale in panels a and b is inverted compared to c and d, so that in all panels red colors correspond to changes caused by increasing temperatures*

3 - Reading the manuscript, I was somewhat disappointed: the summary promises a "novel indicator", but I did not see any novel indicators. I am not sure what the authors are referring to, since neither HDD/CDD nor multi-model means are particularly novel. In the end, I think perhaps it was a poor choice of words in the summary that oversold it a little? If not, could the authors perhaps tell me which of the indicators is supposedly novel?

Response:

We agree with Reviewer #2 that this term was misleading. HDD and CDD are established and widely used indicators of energy demand. Our novel approach consists in investigating climate-driven trends in energy demand for heating and cooling buildings at the global scale, including their uncertainties. We achieve this by using temperature-based indicators, which are calculated as annual sums of HDD or CDD, averaged over several 20-year time periods in the past and in the future. Climate-driven energy-demand trends are then studied based on the absolute and relative differences in these proxies between the time periods.

Changes in the manuscript:

We have replaced the term "novel indicator" in the abstract by:
"**Using the heating and cooling degree-days methodology for thirty global climate model simulations**" (L27).

Furthermore, we define more clearly our proxies in the first subsection of the *Results:*

Proxies of climate-driven energy demand
HDD and CDD calculated with the temperature of historical climate simulations have been validated against observations(Petri and Caldeira, 2015; Spinoni et al., 2018). We define our heating and cooling - climate-driven energy demand - proxies as the annual HDD and CDD sums calculated from daily mean, minimum and maximum temperatures following the UK Met Office methodology (Table 1, Methods section) for each of the 30 CMIP5 climate simulations (Table S2). The advantage of HDD or CDD annual sums is that they can be compared on a global scale, regardless of the timing and length of local heating and cooling seasons. The heating and cooling proxies are presented for the MMM as averages over three 20-year periods 1941-1960, 1981-2000 and 2021-2040 (Fig. S1).

4 - Finally, I suggest revising the manuscript with a stronger focus on clarity. Many sentences are very involved -- especially some of the "elevator analysis" sentences that present increases and decreases -- and I had to read them several times before understanding what quantities were actually increasing and decreasing. I think potential future readers would greatly appreciate it if the authors could revise the manuscript with this in mind.

Response:
We thank Reviewer #2 for pointing out these shortcomings. We have thoroughly revised the manuscript focusing on language and the clarity of the sentences, especially regarding the presentation of increasing and decreasing values in the sections "Heating and cooling changes in the past and in the future", "Comparing trends in heating and cooling" and "Uncertainty from inter-model variability". We paid special attention to simplifying or splitting long and complex sentences. We now use consistent wording to refer to the decrease in the heating proxy and to the increase in the cooling proxy, weak(er)/strong(er) trends and small(er)/large(er) variability.

Changes in the manuscript:

For example, the paragraph (L143-150 of the previous version):
The decreasing trend in heating for the MMM ranged from -20% to 0% in the past and was spatially uniform, especially over continental areas (Fig. 1a). This trend is projected to be even more pronounced in the future, ranging from -80% to -5% (Fig. 1b). The increasing trend in cooling for the MMM ranged between 0% to +20% over continental areas in the past (Fig. 1c), while the trend in cooling for the future exceeds +10% everywhere. This trend in cooling is projected to reach at least +20% over temperate regions, and more than +60% in many northern hemisphere regions (Fig. 1d). Over mid-latitude oceans, the projected trend in cooling is to exceed +100%, which leads to strong gradients close to the coastlines.

has been modified into (L143-151):
Over continental areas, the decreasing trend in the MMM heating proxy was weak, ranging from -20% to 0% in the *past* (Fig. 2a). This trend is projected to become clearly negative everywhere in the *future*, reaching at least -5% (Fig. 2b).
The increasing trend in the MMM cooling proxy was weak in the *past,* ranging between 0% to +20% over continental areas (Fig. 2c). This trend is also projected to be more pronounced in the *future*, exceeding +10% everywhere, reaching at least +20% over mid-latitude regions, and more than +60% in many northern hemisphere regions (Fig. 2d). Over mid-latitude oceans, the projected trend in the cooling proxy is to exceed +100%, which leads to strong gradients close to the coastlines, where an important part of the population lives.

Further language-related changes are highlighted in the manuscript.

References cited in the replies to the reviewers

Apadula, F., Bassini, A., Elli, A. and Scapin, S.: Relationships between meteorological variables and monthly electricity demand, Appl. Energy, 98, 346–356, doi:10.1016/j.apenergy.2012.03.053, 2012.

Borah, P., Singh, M. K. and Mahapatra, S.: Estimation of degree-days for different climatic zones of North-East India, Sustain. Cities Soc., 14(1), 70–81, doi:10.1016/j.scs.2014.08.001, 2015.

Bréon, F.-M., Boucher, O. and Brender, P.: Inter-annual variability in fossil-fuel CO 2 emissions due to temperature anomalies, Environ. Res. Lett., 12(7), 074009, doi:10.1088/1748-9326/aa693d, 2017.

De Cian, E. and Sue Wing, I.: Global Energy Consumption in a Warming Climate, Environ. Resour. Econ., 72(2), 365–410, doi:10.1007/s10640-017-0198-4, 2019.

Day, T.: Degree-days: theory and application, The Charte., London., 2006.

Isaac, M. and van Vuuren, D. P.: Modeling global residential sector energy demand for heating and air conditioning in the context of climate change, Energy Policy, 37(2), 507–521, doi:10.1016/j.enpol.2008.09.051, 2009.

Maia-Silva, D., Kumar, R. and Nateghi, R.: The critical role of humidity in modeling summer electricity demand across the United States, Nat. Commun., 11(1), 1–8, doi:10.1038/s41467-020-15393-8, 2020.

Mukherjee, S., Vineeth, C. R. and Nateghi, R.: Evaluating regional climate-electricity demand nexus: A composite Bayesian predictive framework, Appl. Energy, 235(April 2018), 1561–1582, doi:10.1016/j.apenergy.2018.10.119, 2019.

Nateghi, R. and Mukherjee, S.: A multi-paradigm framework to assess the impacts of climate change on end-use energy demand, edited by X. Hu, PLoS One, 12(11), e0188033, doi:10.1371/journal.pone.0188033, 2017.

Petri, Y. and Caldeira, K.: Impacts of global warming on residential heating and cooling degree-days in the United States, Sci. Rep., 5, 1–14, doi:10.1038/srep12427, 2015.

Spinoni, J., Vogt, J. V., Barbosa, P., Dosio, A., McCormick, N., Bigano, A. and Füssel, H. M.: Changes of heating and cooling degree-days in Europe from 1981 to 2100, Int. J. Climatol., 38(December 2017), e191–e208, doi:10.1002/joc.5362, 2018.

Steemers, K. and Yun, G. Y.: Household energy consumption: a study of the role of occupants, Build. Res. Inf., 37(5–6), 625–637, doi:10.1080/09613210903186661, 2009.

Thom, E. C.: The Discomfort Index, Weatherwise, 12(2), 57–61, doi:10.1080/00431672.1959.9926960, 1959.

Thom, H. C. S.: The rational relationship between heating degree days and temperature, Mon. Weather Rev., 82(1), 1–6, doi:10.1175/1520-0493(1954)082<0001:TRRBHD>2.0.CO;2, 1954.

Trotter, I. M., Bolkesjø, T. F., Féres, J. G. and Hollanda, L.: Climate change and electricity demand in Brazil: A stochastic approach, Energy, 102, 596–604, doi:10.1016/j.energy.2016.02.120, 2016.

Urge-Vorsatz, D., Petrichenko, K., Staniec, M. and Eom, J.: Energy use in buildings in a long-term perspective, Curr. Opin. Environ. Sustain., 5(2), 141–151, doi:10.1016/j.cosust.2013.05.004, 2013.

Valor, E., Meneu, V. and Caselles, V.: Daily Air Temperature and Electricity Load in Spain, J. Appl. Meteorol., 40(8), 1413–1421, doi:10.1175/1520-0450(2001)040<1413:DATAEL>2.0.CO;2, 2001.

Wang, H. and Chen, Q.: Impact of climate change heating and cooling energy use in buildings in the United States, Energy Build., 82(2014), 428–436, doi:10.1016/j.enbuild.2014.07.034, 2014.

Wenz, L., Levermann, A. and Auffhammer, M.: North–south polarization of European electricity consumption under future warming, Proc. Natl. Acad. Sci., 114(38), E7910–E7918, doi:10.1073/pnas.1704339114, 2017.

Reviewer comments, second round –

Reviewer #1 (Remarks to the Author):

The authors have done a commendable job in responding to all my queries. I believe the manuscript quality has significantly increased. One suggestion I have is introducing the "climate-driven" term in the title. May be changing the title to: "Large uncertainties in climate-driven energy demand trends for heating and cooling under climate change". However, I leave the choice to the authors.

Reviewer #2 (Remarks to the Author):

The authors appear to have addressed all my initial concerns satisfactorily, and the quality of the manuscript has definitely improved.

However, I still have some minor concerns and specific corrections that I would like the authors to consider before publication:
- l. 51, "more precisely its combination with humidity": Humidity and wind?
- ll. 56-57, "This minimum is found for a similar daily mean temperature around 16 C, even over a large area like Europe.": I did not understand what is meant by this statement.
- l. 85, "estimate energy demand": The paper does not actually present estimates of energy demand, but estimates of some of the main determinants of energy demand (HDD and CDD).
- l. 187, "climate-driven energy demand": Again, the paper does not present estimates of energy demand, but estimates of determinants of energy demand.
- l. 188, "anthropogenic emission pathways and f climate projections": What does the "f" mean?
- ll. 288-298: If the UK method is the main focus, perhaps the UK method should be discussed first in this paragraph?
- l. 339, "climate-driven trends in the energy demand": Once again, the paper is not really presenting energy demand, but determinants or drivers of energy demand.
- ll. 340-342: I found it difficult to understand what was done.
- ll. 354-355: I know that this is discussing supplementary material, but I am not sure what the point of this particular exercise is -- it doesn't make much sense to me.
- ll. 340-355: I suggest briefly including the reason for performing each of these analyses. What is the point of each of them?
- ll. 362-364: According to the text, it sounds like a t-test was used to tell if the number of HDDs or CDDs were different between two time periods. But the underlying distribution of HDDs and CDDs is not really expected to be normal, because HDDs and CDDs are left-censored: they are never below 0. I suggest revisiting the use of this test. (Perhaps performing the t-test on raw temperatures instead of HDDs/CDDs?)

Ian M. Trotter

REVIEWERS' COMMENTS

Reviewer #1 (Remarks to the Author):

The authors have done a commendable job in responding to all my queries. I believe the manuscript quality has significantly increased. One suggestion I have is introducing the "climate-driven" term in the title. May be changing the title to: "Large uncertainties in climate-driven energy demand trends for heating and cooling under climate change". However, I leave the choice to the authors.

We thank the reviewer for their positive opinion on the revised version that we proposed.

We prefer to leave the title as it is currently because we consider that it makes a redundancy with the terms “climate-driven” and “under climate change”. Nevertheless, the reader must clearly understand that this study deals only with the climate-driven energy demand trends at the global scale. It is why this term appears often in all sections of the manuscript (abstract, introduction, results, and conclusion).

Reviewer #2 (Remarks to the Author):

The authors appear to have addressed all my initial concerns satisfactorily, and the quality of the manuscript has definitely improved.

We thank the reviewer for his kind comment concerning the revised version.

However, I still have some minor concerns and specific corrections that I would like the authors to consider before publication:
- l. 51, "more precisely its combination with humidity": Humidity and wind?

We agree with the reviewer that there are other relevant variables to study the climate-driven energy demand, which are listed in the discussion (l. 246). However temperature is the main climatic driver of the energy demand. In the study of Mukherjee et al. (2019) that we cite to justify this statement, the dew point temperature appears to be the best predictor. Wind is the second but this study shows that it is highly dependent on the US state considered.

Because humidity modulates the diurnal cycle of temperature, the dew point temperature, which is a combination of temperature and humidity (it is approximated by the relation: $Td = T - (100 - RH) / 5$), is better than the ambient temperature.

- ll. 56-57, "This minimum is found for a similar daily mean temperature around 16 C, even over a large area like Europe.": I did not understand what is meant by this statement.

We have reformulated this sentence. The sentence: “This minimum is found for a similar daily mean temperature around 16 °C, even over a large area like Europe ”

has been changed to:

“This minimum is found for a similar daily mean temperature around 16 °C for 35 countries in Europe”

- l. 85, "estimate energy demand": The paper does not actually present estimates of energy demand, but estimates of some of the main determinants of energy demand (HDD and CDD).

We agree with the reviewer that we are not directly investigating the climate-driven energy demand because we are using proxies derived from the Degree-Days method.
In this sentence, we have added the term “proxies” (in bold) such as:

”This study focuses on **proxies** of the climate-driven energy demand for heating and cooling buildings, and presents the first global analysis of future trends together with a comprehensive analysis of uncertainties linked to temperature projections.”

- l. 187, "climate-driven energy demand": Again, the paper does not present estimates of energy demand, but estimates of determinants of energy demand.

We agree again with the reviewer.

- l. 188, "anthropogenic emission pathways and f climate projections": What does the "f" mean?

We thank the reviewer to spotting this typo.

This remark and the previous one concern the same sentence. This sentence has been modified from:
“The wide range of projections of future climate-driven energy demand for heating and cooling buildings stems from both the uncertainty of anthropogenic emission pathways and f climate projections.”

to:

”The uncertainty of anthropogenic emission pathways and of climate projections both contribute to the wide range of projections of future climate-driven energy demand for heating and cooling buildings.”

- ll. 288-298: If the UK method is the main focus, perhaps the UK method should be discussed first in this paragraph?

We think that it is easier to understand the interest of the UK method that we mostly use after introducing the US method because the latter method is less elaborated.

- l. 339, "climate-driven trends in the energy demand": Once again, the paper is not really presenting energy demand, but determinants or drivers of energy demand.

Once again we agree with the reviewer. We have carefully examined the entire manuscript to precise that we are investigating the climate-driven energy demand through proxies.

In this precise sentence, we have added the term “proxy” (in bold) as follows:
“In addition to the general methodology described by the five steps above, we list particular alternative methods below that have been used to test the robustness **of the proxies** of climate-driven trends in the energy demand”

- ll. 340-342: I found it difficult to understand what was done.

In order to clarify what is done, we have modified the sentence:
“We use the same methodology (modifying Steps 2, 3, 4 and 5) with annual average of surface air temperature for the three time periods (Fig. S1g-i) in order to compare the spatial patterns with the ones of HDD and CDD in Supplementary Material Section I.”

To:

“In order to compare the spatial patterns of HDD and CDD with the one of temperature, we use the same methodology with annual averages of surface air temperature for the three time periods (Fig. S1g-i) in order to calculate the MMM of surface air temperature in Supplementary Material Section I.”

- ll. 354-355: I know that this is discussing supplementary material, but I am not sure what the point of this particular exercise is -- it doesn't make much sense to me.

We do not consider this test as a useful alternative to our general methodology, however this is often used in climate studies (for example focusing on glaciers). We have changed the sentence:

“We calculate HDD and CDD from a multi-model mean of daily temperature (modifying Step 1) in Supplementary Material Section IIIe.”

To:

“To test the influence of applying the multi-model mean on daily temperature instead of on HDD and CDD, we calculate HDD and CDD from a multi-model mean of daily temperature (modifying Step 1) in Supplementary Material Section IIIe. Note that this method leads to a single value of HDD and CDD (instead of the 30 for the other tests).”

In Supplement (Section IIIe), a sentence has been added (in yellow below):

"Still, it is advised to work with temperature simulations of all available climate models rather than multi-model temperatures to provide a range of possible future temperatures for calculating degree days in the context of energy demand predictions. Although this methodology leads to similar simulated trends in the heating and cooling proxies, it hides the uncertainties due to the large inter-model variability."

- ll. 340-355: I suggest briefly including the reason for performing each of these analyses. What is the point of each of them?

A sentence has been added for each of the five tests (in yellow below corresponding to l. 344-366 of the revised version):

"In addition to the general methodology described by the five steps above, we list particular alternative methods below that have been used to test the robustness of the proxies of climate-driven trends in the energy demand:

a) To test if the results are influenced by the Degree-Days calculation method, we compare the two most widely used approaches, the UK Met Office calculation (applied to obtain our results presented in the main article) and a simpler calculation used in the USA. We use HDD and CDD with US ASHREA (American Society of Heating, Refrigerating and Air-Conditioning Engineers) equations (Table 2) instead of the UK Met Office (modifying Step 1) in Supplementary Material Section IIIa.

b) To test the influence of the spatial interpolation, we reduce the spatial resolution of the grid from 1° by 1° (applied to obtain our results presented in the main article) to 2° by 2°. We interpolate on a 2° grid (modifying Step 4) in Supplementary Material Section IIIb.

c) To test the influence of the length of the time span over which HDD and CDD are averaged, we increase the length of the time spans over which annual HDD and CDD are averaged from 20 years (applied to obtain our results presented in the main article) to 30 years in Supplementary Material Section IIIc.

d) To test the influence of the systematic biases that appears in some regions for some models compared to observations, we correct the model biases based observations of the CRU TS 4.0 data set at 0.5° resolution[29] in Supplementary Material Section IIId.

e) To test the influence of applying the multi-model mean on daily temperature instead of on HDD and CDD, we calculate HDD and CDD from a multi-model mean of daily temperature (modifying Step 1) in Supplementary Material Section IIIe. Note that this method leads to a single value of HDD and CDD (instead of the 30 for the other tests).

- ll. 362-364: According to the text, it sounds like a t-test was used to tell if the number of HDDs or CDDs were different between two time periods. But the underlying distribution of HDDs and CDDs is not really expected to be normal, because HDDs and CDDs are left-censored: they are never below 0. I suggest revisiting the use of this test. (Perhaps performing the t-test on raw temperatures instead of HDDs/CDDs?)

Daily HDD and CDD are left-censored, but for specific locations (i.e. grid cell), the cumulative HDD or CDD over a year will be comparable from one year to another. Consequently we expect a normal distribution over a long time period. As we compare the averages of the 30 CMIP5 for two different time periods, we assume that these values are normally distributed.

In the Method section (l. 369-376 of the revised version), we have added information (in yellow below).

"The absolute differences in HDD and CDD values (i.e. the annual HDD and CDD sums averaged over 20 years) are calculated between the 1981-2000 and 1941-1960 averages, referred to as past changes, and between the 2021-2040 and 1981-2000 averages, referred to as future changes, for each model and for the MMM (Fig. 1). Heating and cooling trends are expressed in terms of relative HDD and CDD differences, in percentage compared to the earlier period, shown for the MMM (Fig. 2). A Student t-test (using two dependent data samples) is applied to test whether the averages of the thirty simulations are significantly different over two compared time periods at a 95% confidence interval, assuming that the values of the thirty simulations are normally distributed in each grid cell."

References:

Mukherjee, S., Vineeth, C. R. & Nateghi, R. Evaluating regional climate-electricity demand nexus: A composite Bayesian predictive framework. *Appl. Energy* **235**, 1561–1582 (2019)

Response To Reviewers Letter

Dear Reviewers:

Thank you for the constructive comments concerning our manuscript entitled "TNF-α-mediated m^6A modification of ELMO1 triggers directional migration of mesenchymal stem cell in ankylosing spondylitis" (Manuscript ID: NCOMMS-20-35339A). These comments were valuable and very helpful for improving our manuscript to better demonstrate the important significance of our research. We have carefully reviewed all comments and completed point-by-point revisions. The responses to the comments in this letter and the revised portions of the manuscript are marked in red. We appreciate the work of Reviewers and hope that the revisions will meet with approval. We will be glad to respond to any further comments that you may have.

Yours sincerely,

Huiyong Shen

Department of Orthopedics, The Eighth Affiliated Hospital, Sun Yat-sen University, 3025# Shennan Road, Shenzhen, 518000, P.R. China.

Tel: +86 139 2227 6368

Fax: +86 20 8133 2612

Email: shenhuiy@mail.sysu.edu.cn

Responses to Reviewer 1

The authors have significantly improved their manuscript and provide thoughtful answers to previous criticisms. The paper is more focused without the filiopodia claims. Also, the paper is significantly improved with the in vivo data that Curdulan upregulates Elmo1 expression. The mechanims of MTTL4 stability and the RNA-seq data adds to the paper and reveal the complexity of the system. While pleotropic effects are observed, Elmo1 is clearly a target. I think these studies add to the manuscript and conclusions.

While the novelty on Elmo in migration remains a limitation of the study, this is now well balanced with the added new experiments and the global conclusions.

Minor comment #1. I still find that it is irrelevant to show co-ip of Elmo1 with Dock1, Dock4 and Dock5, but I leave it up to the authors. The new Rac pull-down data is very convincing - congrats to the authors.

Response: Thank you very much for the comment. Due to the results of Co-IP and LC-MS/MS, we chose to retain the data of DOCK1, 4 and 5 in our manuscript.

Minor comment #2. The authors should introduce the Chang L. 2020 (Nat Comms) when discussing the mechanisms of Elmo-Dock interaction and activation of the complex for Rac activation.

Response: Thank you very much for the comment. The mention reference has been added in the Discussion part.

Reviewer #2 (Remarks to the Author):

The authors have adequately addressed my concerns and queries.

Reviewer #3 (Remarks to the Author):

The authors have fully addressed my questions.

Reviewer #4 (Remarks to the Author):

I have no remarks for the revised version of the manuscript by Xie et al " TNF-α-mediated m6A modification of ELMO1 triggers directional migration of mesenchymal stem cell in ankylosing spondylitis". The authors have properly and fully addressed all the questions raised during the first revision.

No response needed.
